# Time-series transcriptome of *Brachypodium distachyon* during bacterial flagellin-induced pattern-triggered immunity

**DOI:** 10.3389/fpls.2022.1004184

**Published:** 2022-09-15

**Authors:** Tsubasa Ogasahara, Yusuke Kouzai, Megumi Watanabe, Akihiro Takahashi, Kotaro Takahagi, June-Sik Kim, Hidenori Matsui, Mikihiro Yamamoto, Kazuhiro Toyoda, Yuki Ichinose, Keiichi Mochida, Yoshiteru Noutoshi

**Affiliations:** ^1^ Graduate School of Environmental and Life Science, Okayama University, Okayama, Japan; ^2^ Bioproductivity Informatics Research Team, RIKEN Center for Sustainable Resource Science, Yokohama, Japan; ^3^ Kihara Institute for Biological Research, Yokohama City University, Yokohama, Japan; ^4^ School of Information and Data Sciences, Nagasaki University, Nagasaki, Japan

**Keywords:** *Brachypodium distachyon*, monocotyledonous plant, microbe-associated molecular pattern, time-series transcriptome analysis, reactive oxygen species, pattern-triggered immunity

## Abstract

Plants protect themselves from microorganisms by inducing pattern-triggered immunity (PTI) *via* recognizing microbe-associated molecular patterns (MAMPs), conserved across many microbes. Although the MAMP perception mechanism and initial events during PTI have been well-characterized, knowledge of the transcriptomic changes in plants, especially monocots, is limited during the intermediate and terminal stages of PTI. Here, we report a time-series high-resolution RNA-sequencing (RNA-seq) analysis during PTI in the leaf disks of *Brachypodium distachyon*. We identified 6,039 differentially expressed genes (DEGs) in leaves sampled at 0, 0.5, 1, 3, 6, and 12 hours after treatment (hat) with the bacterial flagellin peptide flg22. The k-means clustering method classified these DEGs into 10 clusters (6 upregulated and 4 downregulated). Based on the results, we selected 10 PTI marker genes in *B. distachyon*. Gene ontology (GO) analysis suggested a tradeoff between defense responses and photosynthesis during PTI. The data indicated the recovery of photosynthesis started at least at 12 hat. Over-representation analysis of transcription factor genes and cis-regulatory elements in DEG promoters implied the contribution of 12 WRKY transcription factors in plant defense at the early stage of PTI induction.

## Introduction

Plants recognize microorganisms and protect themselves from their invasion by inducing defense responses (Chisholm et al., 2006; Jones and Dangl, 2006). When microbes encounter plants, their cell walls or associated proteins such as flagellum are degraded by lytic enzymes or proteases stored in plant cell walls and apoplastic space. During this process, degradants such as chitin (in fungal cell walls), peptidoglycans (PGNs), lipopolysaccharides (in bacterial cell walls), and a bacterial flagellin peptide are perceived by sensor molecules deployed on the plant’s plasma membrane. This receptor complex includes transmembrane- or membrane-anchored proteins possessing a kinase domain and activates downstream components *via* phosphorylation to trigger defense responses (Boutrot and Zipfel, 2017). Plants can recognize the well-conserved microbial molecules designated as microbe-associated molecular patterns (MAMPs), which enables them to respond to a broad range of microbes in nature (Boller and Felix, 2009). This plant defense response is pattern-triggered immunity (PTI), given the underlying biological process. During PTI, the membrane-localized enzyme NADPH oxidase, also known as respiratory burst oxidase homolog (RBOH), generates reactive oxygen species (ROS). The ROS function as molecules that attack microbes and second messengers that mediate cellular signal transduction (Segonzac and Zipfel, 2011). This leads to the induction of defense-related genes, which produces antimicrobial proteins and chemical compounds.

*Arabidopsis thaliana* recognizes chitin oligomers *via* a receptor complex comprising receptor-like kinases (RLKs), AtCERK1, and AtLYK5, which possess the LysM domain (Miya et al., 2007; Wan et al., 2008; Cao et al., 2014). In rice (*Oryza sativa* L.), the chitin sensor is composed of an RLK (OsCERK1) and a membrane-anchored LysM domain-containing sensor without an intracellular kinase domain (CEBiP) (Kaku et al., 2006; Kishimoto et al., 2010; Shimizu et al., 2010; Liu, T. et al 2012; Hayafune et al., 2014; Kouzai et al., 2014a, 2014b). Both sensor complexes phosphorylate downstream receptor-like cytoplasmic kinase (RLCK) VII members that directly bind to and activate mitogen-activated protein kinase proteins to transduce a signal to cascades composed of MAPKs (Yamaguchi et al., 2013; Ao et al., 2014; Yamada et al., 2016; Li et al., 2017; Bi et al., 2018; Rao et al., 2018). Arabidopsis perceives flg22, an epitope of the N-terminal part of flagellin protein fliC, *via* the RLK sensor complex, which consists of FLS2 and BAK1 (Zipfel et al., 2004; Chinchilla et al., 2007; Sun et al., 2013). RLCK VII subgroups 5, 7, and 8, including BOTRYTIS-INDUCED KINASE1 (BIK1), play an important role in downstream signaling, activating the MAPK cascade (Lu et al., 2010; Laluk et al., 2011; Bi et al., 2018; Rao et al., 2018). BIK1 also activates RBOH *via* phosphorylation to induce ROS production (Kadota et al., 2014; Li et al., 2014). Solanaceous plant species can recognize flg22; however, a subset of these species, including tomato (*Solanum lycopersicum*), potato (*Solanum tuberosum*), and pepper (*Capsicum annuum*), but not tobacco (*Nicotiana* spp.), can also perceive a different flagellin epitope, i.e., flgII-28 (Cai et al., 2011; Clarke et al., 2013). In tomatoes, flgII-28 is recognized by FLS3 RLK in a BAK1-dependent manner (Hind et al., 2016). In monocotyledonous plants such as wheat (*Triticum aestivum*), barley (*Hordeum vulgare*), sorghum (*Sorghum bicolor*), and maize (*Zea mays*), leaf disks have been shown to produce ROS in response to flg22 (Proels et al., 2010; Zhang et al., 2017; Samira et al., 2020; Su et al., 2021). Interestingly, rice plants and cultured cells showed a weak response to flg22, although they could recognize flagellin extracted from *Acidovorax avenae*. This would be because of the low-level expression of the functionally verified receptor OsFLS2, a homolog of Arabidopsis FLS2 (Takai et al., 2008). Rice plants perceive PGN, a cell wall component in Gram-positive and Gram-negative bacteria, *via* the coreceptors OsCERK1 and LYP4 or LYP6, membrane-anchored receptor-like proteins (RLPs) lacking the kinase domain (Liu, B. et al., 2012; Kouzai et al., 2014a). In Arabidopsis, PGN is recognized by a receptor complex consisting of LysM domain-containing RLPs (LYM1 and LYM3) and CERK1 (Willmann et al., 2011).

Global gene expression in Arabidopsis has been extensively characterized during PTI. Expression analysis of 8,200 genes in cultured Arabidopsis cells (at 30 and 60 min after treatment with 100 nM flg22) and seedlings (at 60 min after treatment with 10 μM flg22) by microarray resulted in the identification of 225 and 252 differentially expressed genes (DEGs), respectively (Navarro et al., 2004). Further microarray analysis of 23,000 genes in Arabidopsis seedlings at 30 min after treatment with flg22 (10 µM) revealed 966 upregulated and 202 downregulated genes (Zipfel et al., 2004). Gene expression has also been examined in Arabidopsis mesophyll protoplasts and seedlings at 30 and 60 min after treatment with flg22 (100 nM and 2 nM, respectively) using ATH1 GeneChip arrays (Boudsocq et al., 2010). Additionally, Arabidopsis gene expression in response to 100 μg/ml PGN or 1 μM flg22 was analyzed by microarray at 4 h post-treatment, and 236 genes were found to be upregulated by PGN, many of which were also responsive to flg22 (Gust et al., 2007). Nine of these genes were analyzed for transcriptional changes at 1, 2, 4, 6, and 24 h, and their return to steady-state levels at 24 h was detected as a reaction termination. Winkelmuller et al. (2021) performed RNA-seq analysis of the seedlings of six Arabidopsis accessions and three Brassicaceae species, *C. rubella* (Cru), *C. hirsuta* (Chi), and *E. salsugineum* (Esa), at 1, 9, and 24 h after the treatment with flg22 (1 μM). They identified 868 genes commonly upregulated at 1 h in all samples. The restoration of these increased expressions occurred at 24 h. A recent study identified core genes commonly induced by various MAMPs and abiotic stresses within 3 h, and these genes were proposed to be a part of the plant general stress response (GSR) (Bjornson et al., 2021).

Compared with Arabidopsis, transcriptional analyses in monocotyledonous plants during PTI are limited. Fujiwara et al. (2004) reported transcriptome analysis of cultured rice cells at 0.5, 1, 2, 3, 4, 5, and 6 h after the treatment with *A. avenae* compatible and incompatible strains using a microarray consisting of 3,353 rice cDNA clones which identified 131 DEGs. Thus, PTI in monocotyledonous plants have not yet been characterized with high-resolution transcriptome analysis and its details were not elucidated; for example, how many genes were affected, how their transcription altered, and when and how the transcriptional changes terminated.

Here, we report a time-course analysis of MAMP-induced transcriptomic changes in the leaf disks of the model monocot *Brachypodium distachyon*. Unlike rice, *B. distachyon* showed a response to flg22 treatment and produced massive ROS. At 0,5 –12 hours after treatment with flg22 (hat), a total of 6,039 DEGs were identified. These genes could be divided into 10 clusters, including 4 downregulated clusters (2,494 DEGs) and 6 upregulated clusters (3,545 DEGs). The expression of photosynthesis-related genes was reduced, whereas that of defense-related genes was induced during PTI. DEGs related to photosynthesis or chloroplast function were enriched in cluster 2 (with expression downregulated at 3 h but recovered at 12 h) and cluster 10 (with expression upregulation at 12 h), suggesting that the recovery from defense responses at the transcriptional level occurs at the least at 12 hat. Analysis of the correlation between the induced transcription factor (TF) genes and DEGs harboring cis-regulatory elements in their promoters implied the importance of WRKY TFs for PTI induction.

## Materials and methods

### Plant materials and MAMPs

*Brachypodium distachyon* accession Bd21 was obtained from the USDA-ARS National Plant Germplasm System. Dry seeds were sterilized and then incubated for 7 days in a plastic Petri dish lined with a moistened filter paper to induce germination. The seedlings were transferred to soil (Sakata Supermix-A; Sakata Seed, Yokohama, Japan) and grown for 2 weeks under light-emitting diodes (LEDs) in a growth chamber (LPH-350S; Nippon Medical & Chemical Instruments, Osaka, Japan) maintained at 25°C and 16 h light/8 h dark photoperiod. The plants were regularly watered with a 1,000-fold diluted fertilizer (Professional hyponex 10-30-20; Hyponex, Osaka, Japan). Synthetic flg22 peptide, purified chitinheptaose, and PGN from *Bacillus subtilis* was purchased from KareBay Biochem, Inc. (KP0745, Monmouth Junction, NJ, USA), ELICITYL (GLU437, Crolles, France), and Sigma-Aldrich (69554, St. Louis, MI, USA), respectively. Dr. Hironori Kaminaka at Tottori University kindly gifted chitin nanofiber.

### ROS measurement

Leaf disks were excised from the topmost fully expanded leaf of 4–5-week-old *B. distachyon* plants using a disposable biopsy punch (BPP-30F, φ3.0 mm; Kai Industries, Seki, Japan). The leaf disks floated on sterilized distilled water in a Petri dish covered with a lid and incubated at room temperature for 8 h. Luminol solution (10 μl of 2 mM L-012 [120-04891; FUJIIFLM Wako Pure Chemical Corp., Osaka, Japan], 10 μl of 100 μg/ml horseradish peroxidase [P8125; Sigma-Aldrich], and 70 μl water) was added into each well of a white 96-well microplate (655904; Greiner Bio-One, Frickenhausen, Germany). Leaf disks were placed on the luminol solution in the well and incubated for 1 h in the dark. After verifying that the level of background luminescence was stable, different concentrations of MAMPs (10 μl) were added to the wells, and luminescence was measured over time using a microplate reader (TriStar2 LB942; Berthold Technologies, Bad Wildbad, Germany).

### RNA-seq analysis

*B. distachyon* leaf disks sampled at the indicated time points after treatment with flg22 were frozen in liquid nitrogen and crushed with a beads cell disrupter (MS-100; TOMY SEIKO, Osaka, Japan) and zirconia balls (YTZ-3; Nikkato, Osaka, Japan). Total RNA was extracted with three biological replicates from the homogenized leaf tissue using the Pure Link RNA Mini Kit (Thermo Fisher Scientific, Waltham, MA, USA). All subsequent steps, including RNA quality analysis using 2100 Bioanalyzer (Agilent, Santa Clara, CA, USA), library preparation, RNA-seq with BGISEQ, and read trimming, were performed by BGI (New Territories, Hong Kong).

The quality of RNA-seq data was checked by FastQC (https://www.bioinformatics.babraham.ac.uk/projects/fastqc/), and unpaired sequence reads were removed using Trimmomatic (version 0.39) (Bolger et al., 2014). The total number of paired-end reads obtained in this study and the selection results of this step are summarized in **Supplementary Table S1**. The remaining paired sequence reads were mapped to the *Brachypodium distachyon* v3.1 reference genome sequence retrieved from Phytozome using STAR (version 2.7) (Dobin et al., 2013). These mapping results are summarized in **Supplementary Table S2**. By using these mapped reads, gene expression levels were estimated with RSEM v1.3.2 (Li and Dewey, 2011). The count data for each gene of all samples is shown in **Supplementary Table S3**. Genes showing significant differences in average expression levels over three replicates between any of the time points were extracted by edgeR (version 3.24.3) with the quasi-likelihood method (**Supplementary Table S4**), and then differentially expressed genes (DEGs) showing more than a 2-fold increase or decrease (FDR < 0.01, *p* < 0.01) were extracted with Benjamini & Hochberg (BH) method (**Supplementary Table S5**) (Robinson et al., 2010). The default parameters were used for each process.

### Z-scaling and k-means clustering of DEGs

The expression levels of DEGs at different time points (0 to 12 hat) were converted into z-scores using the genescale function in the genefilter (version 1.64.0) of Bioconductor R package (Gentleman et al., 2004). Based on z-scores, the DEGs were classified into 10 clusters using a k-means clustering algorithm in Multi Experiment Viewer (MeV) (Saeed et al., 2006).

### qRT-PCR analysis

The cDNAs of 12 selected genes were synthesized using PrimeScript RT Reagent Kit with gDNA Eraser (Takara Bio, Kusatsu, Japan). Then, qPCR was performed on the LightCycler 96 Real-Time PCR System (Roche, Pleasanton, CA, USA) using Luna Universal qPCR Master Mix (New England Biolabs, Ipswich, MA, USA) and sequence-specific primers (**Supplementary Table S9**) designed using the Primer3 online software tool (Untergasser et al., 2012). The expression of each gene was analyzed in biological replicates. *BdUbi4* was used for data normalization (Chambers et al., 2012).

### GO enrichment analysis

The closest homologs of *A. thaliana* genes in *B. distachyon* were identified by blastP search (a threshold e-value of < 1e-5) using the deduced amino acid sequences. GO analysis of *B. distachyon* DEGs in each cluster was based on Arabidopsis gene information using agriGO v2.0 (http://systemsbiology.cau.edu.cn/agriGOv2/) and a list of GO terms with their frequency of occurrence. GOs for photosynthesis/chloroplast-related functions and defense-related functions were extracted (marked with green and yellow color bars, respectively in **Supplementary Table S6-1–S6-10**) and their occurrences in each cluster were depicted as a color-coded graph using MeV; the darker the color, the lower the *p*-value.

### Over-representation analysis of TF genes and their potential downstream genes in each cluster

TF-types of each *B. distachyon* gene shown in column F of **Supplementary Table S7** were retrieved from the PlantTFDB v5.0 website (Jin et al., 2017). Then, the number of DEGs encoding TFs was counted. The TF category and the corresponding number of DEGs examined in this study are as follows: AP2 (24), AP2-ERF (1), ARF (26), ARR-B (16), B3 (51), BBR-BPC (3), BES1 (8), bHLH (146), bZIP (86), C2H2 (96), C3H (47), CAMATA (7), CO-like (13), CPP (9), DBB (9), Dof (29), E2F/DP (11), EIL (6), FAR1 (124), G2-like (51), GATA (29), GeBp (14), GRAS (63), GRF (12), HB-other (13), HB-PHD (3), HD-ZIP (40), HRT-like (1), HSF (24), LBD (28), LFY (1), LSD (5), MIKC_MADS (34), M-type-MADS (45), MYB (117), MYB/MYB-related (6), MYB-related (61), NAC (136), NF-X1 (2), NF-YA (7), NF-YC (16), Nin-like (16), RAV (4), SIFa-like (1), SBP (17), SRS (6), STAT (1), TALE (23), TCP (21), Trihelix (30), VOZ (2), Whirly (2), WOX (13), WRKY (87), YABBY (8), and ZF-HD (21) (**Supplementary Table S7**). The number of TF genes in each cluster was also counted, and the ratio of their abundance to the total number of genes (over-representation) was calculated using Fisher’s hypergeometric test (Tavazoie et al., 1999) using R. The numbers and *p*-values are shown in **Supplementary Table S8**. The data of only AP2/ERF, bHLH, bZIP, MYB, NAC, and WRKY are shown in **Figure 6**.

A 1-kb sequence upstream of the start codon of each gene was first extracted from *B. distachyon* whole-genome data using BEDtools getfasta (version 2.25.0) to perform the over-representation analysis of DEGs harboring particular cis-regulatory elements in their promoter regions (Quinlan and Hall, 2010). Then, using a custom Python script (available through a GitHub repository (https://github.com/junesk9)), the following cis-elements were identified in the extracted promoter regions: GCC-box (AGCCGCC), DRE/CRT ((A/G)CCGAC), and DRE-like ((A/G/T)(A/G)CCGACN(A/T)) for AP2/EFR; Myc-related (CACATG) for MYC/NAC; G-box (CACGTG), G-box-like (CACGT(A/T)), ABRE-like ((C/G/T)ACGTG(G/T)(A/C)), ACTCAT-element (ACTCAT), and TGA-element (TGACG) for bZIP; AtMyb1 ((A/C)TCC(A/T)ACC), AtMyb2 ((A/C)TCC(A/T)ACC), AtMyb3 (TAACTAAC), and AtMyb4 (A(A/C)C(A/T)A(A/C)C) for MYB; W-box (TTGAC/T) for WRKY; and CG-1 ((A/C/G)CGCG(T/C/G)) for CAMTA. The number and abundance ratio of cis-elements in each cluster were also analyzed using Fisher’s hypergeometric test to evaluate over-representation.

## Results

### MAMP response in *B. distachyon* leaf disks

**Figure 1:**
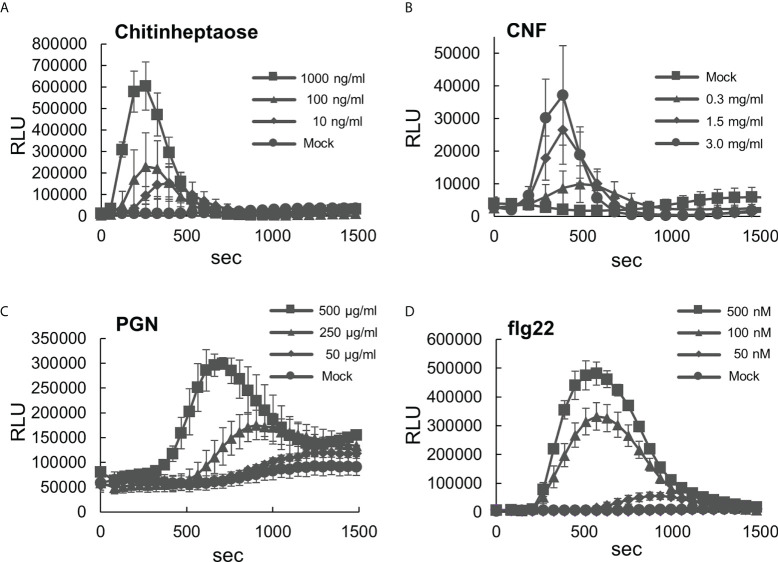
Analysis of reactive oxygen species (ROS) production in the leaf disks of *Brachypodium distachyon* treated with various MAMPs. **(A–D)** Leaf disks of *B distachyon* treated with chitinheptaose **(A)**, flg22 peptide **(B)**, peptidoglycan **(C)**, and chitin nanofiber **(D)**. Fluorescence was measured using a microplate reader and expressed as a relative light unit (RLU). Data represent mean ± standard error (*n* = 4).

To use *B. distachyon* as a model system for PTI analysis in monocots, we initially characterized its responsiveness to various types of MAMPs. First, ROS production in *B. distachyon* leaf disks treated with selected MAMPs was measured using the luminol (L-012)-based assay (Albert et al., 2015). This study used two fungal elicitors, including chitinheptaose (a purified chitin heptamer) and chitin nanofiber prepared from crab shells (Egusa et al., 2015). Transient ROS production peaks at 250–400 s were detected upon treatment with 10, 100, and 1000 ng/ml chitinheptaose in a concentration-dependent manner (**Figure 1A**). *B. distachyon* leaf disks also produced ROS after treatment with 0.3, 1.5, and 3.0 mg/ml chitin nanofiber (**Figure 1B**). However, the ROS production peaks were slightly delayed, and their magnitude was lower than the peaks obtained using chitinheptaose. Additionally, three bacterial elicitors including PGN, flg22 peptide, and elf18 peptide (part of the elongation factor Tu [EF-Tu] protein) were also tested (Kunze et al., 2004). ROS were produced with the PGN (50, 250, and 500 μg/ml) treatment in a concentration-dependent manner, with peaks at 600–1300 s (**Figure 1C**). ROS peaks were also detected between 600 and 1000 s after treatment with 50, 100, and 500 nM flg22 (**Figure 1D**). This is in contrast to the results reported in rice, which showed weak sensitivity to flg22 (Takai et al., 2008). However, no ROS production occurred after the elf18 treatment (**Supplementary Figure S1**). This is consistent with the report that the EF-Tu (ef18) perception is restricted to the Brassicaceae (Boller and Felix, 2009).

### Time-series RNA-seq analysis of *B. distachyon* leaf disks during PTI

**Figure 2:**
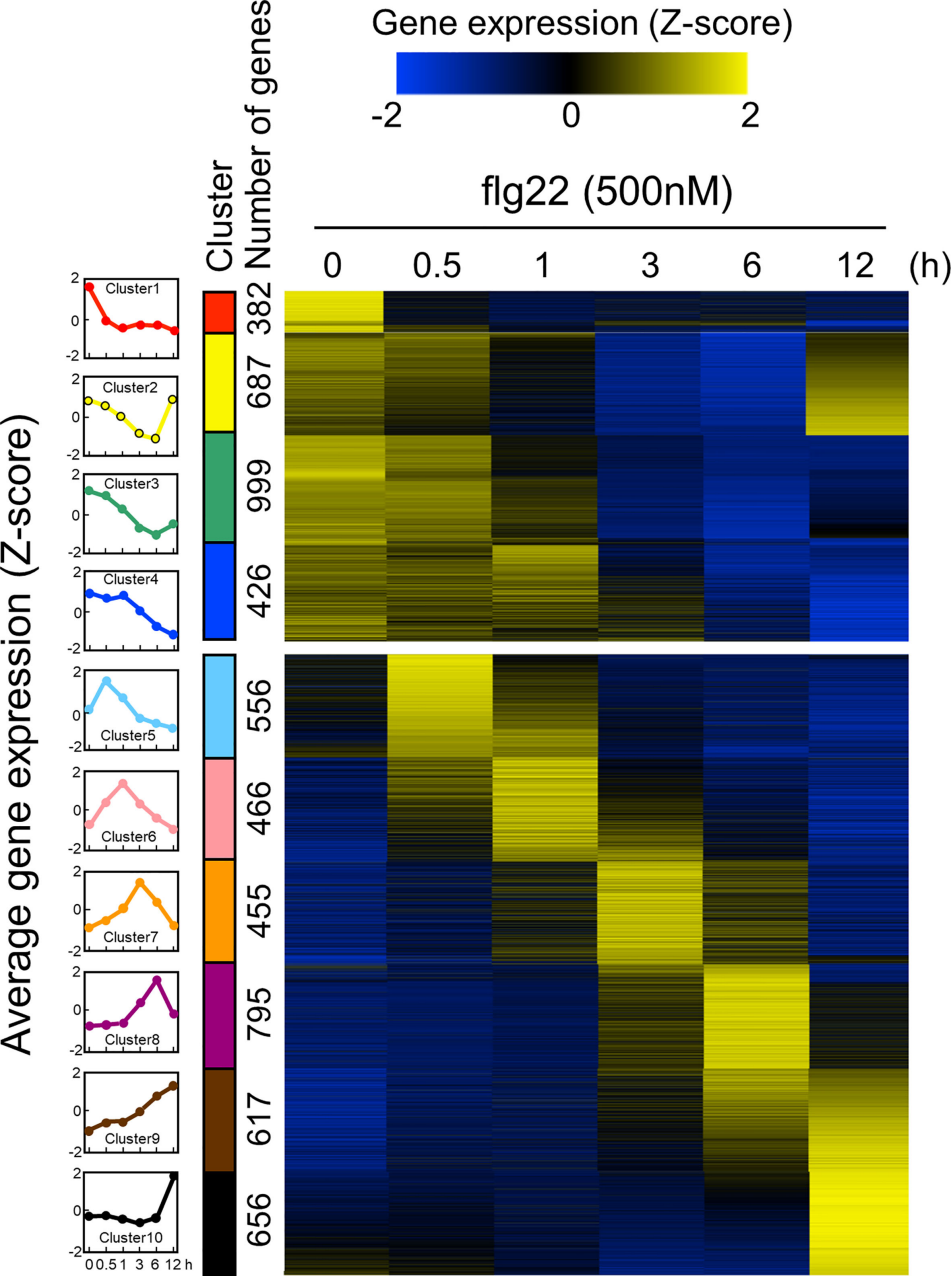
Expression patterns of *Brachypodium distachyon* genes in leaf disks treated with flg22. A total of 6,039 differentially expressed genes (DEGs) were identified by RNA-seq analysis of leaf disks treated with flg22 for the indicated time periods. Expression levels of genes at different time points were converted to z-scores and displayed as a heatmap. As shown in the scale bar, the higher expression values compared with the other time points are represented in yellow color. The flg22-responsive genes were classified into 10 clusters using the k-means clustering method based on their expression patterns. The average expression pattern of each cluster, top 4 and bottom 6 are down- and upregulated genes, respectively, and shown on the left column with different colors. The number of genes in each cluster is shown along with the colored bars.

Since the ROS measurement assay with leaf disks worked well, we decided to use this experimental system to analyze time-series transcriptome during PTI in *B. distachyon*. The PTI transcriptomes have been accumulated in *A. thaliana* particularly using flg22, therefore, flg22 was used in this study to compare the obtained data with previously published results. Total RNA was extracted from three biological replicates of *B. distachyon* leaf disks treated with flg22 (500 nM) for 0, 0.5, 1, 3, 6, and 12 h, and subjected to RNA-seq analysis. The sequence reads from all 18 samples were mapped to the Bd21 reference genome sequence to determine gene expression profiles. Genes showing 2-fold higher or lower expression than the untreated control were selected as DEGs (**Table 1** and **Supplementary Table S4**). In total, 6,039 DEGs were detected (genes appeared in multiple time points were considered as one DEG). The expression level of each DEG during the flg22 treatment was represented as a Z-score (**Supplementary Table S5**), and the DEGs were divided into 10 groups, based on their expression patterns, using a k-means clustering algorithm (**Figure 2**). The gap statistic computation with R (codes: gapStat.R and KmeansGap.R) assessed the numbers of clusters and the goodness of clustering (**Supplementary Figure S3**) (Tibshirani et al., 2001). We generated heatmaps to illustrate the changes in DEGs’ expression levels. Four clusters (clusters 1–4) contained genes whose expression levels decreased during PTI. The expression levels of genes in clusters 2 and 3 were fully or partially recovered at 12 hat. Clusters 5, 6, 7, 8, and 10 consisted of genes whose expression levels were the highest at 0.5, 1, 3, 6, and 12 hat, respectively. Cluster 9 included genes that expressed both at 6 and 12 hat. In this study, we selected the DEGs by comparing the sample at 0 hat to the other time points, therefore, it must be stated that these results include not only genes due to flg22 treatment, but also those due to diurnal cycles.

**Table 1:** Number of differentially expressed genes (DEGs) identified in this study in *Brachypodium distachyon* leaf disks treated with flg22 at the indicated time points.

	0.5 h	1 h	3 h	6 h	12 h
Up	616	804	1133	1426	1367
Down	296	668	1890	2514	2000
Total	912	1472	3023	3940	3367

### Selection of PTI marker genes in *B. distachyon*

**Figure 3:**
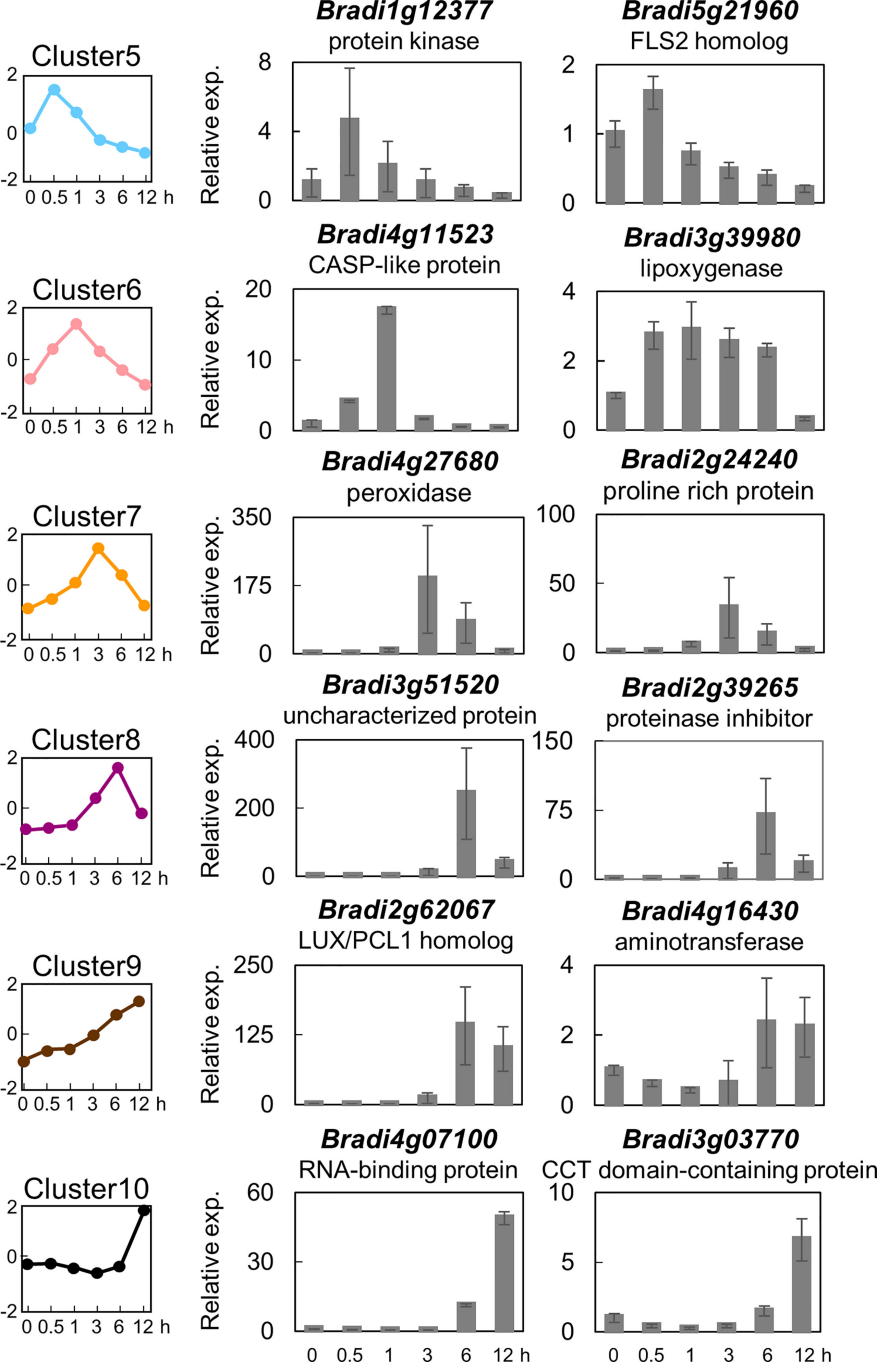
Expression analysis of selected flg22-responsive marker genes of *Brachypodium distachyon* representing the different clusters. Two genes each were selected from clusters 5–10, and their expression was analyzed by qRT-PCR using cDNA prepared from total RNA extracted from *B distachyon* leaf disks sampled at each time point after flg22 (500 nM) treatment. Data represent mean ± SE (*n* = 3).

In order to identify marker genes suitable for monitoring PTI in *B. distachyon*, two genes were selected from six clusters (i.e., clusters 5, 6, 7, 8, 9, and 10), and their expression levels were analyzed by quantitative reverse transcription-polymerase chain reaction (qRT-PCR). As demonstrated in **Figure 3**, the expression patterns of the selected 10 genes were almost similar to the averaged expression profiles of each cluster. These results vouch for the veracity of the RNA-seq experiments. These 10 genes could serve as reliable marker genes for the monitoring of PTI in *B. distachyon*. Among these genes, the amino acid sequence of the protein encoded by *Bradi1g17150* (cluster 5) showed the highest similarity to FLAGELLIN SENSING 2 (FLS2) of Arabidopsis (At5g46330) and rice (LOC_Os04g52780).

### GO enrichment analysis of DEGs

**Figure 4:**
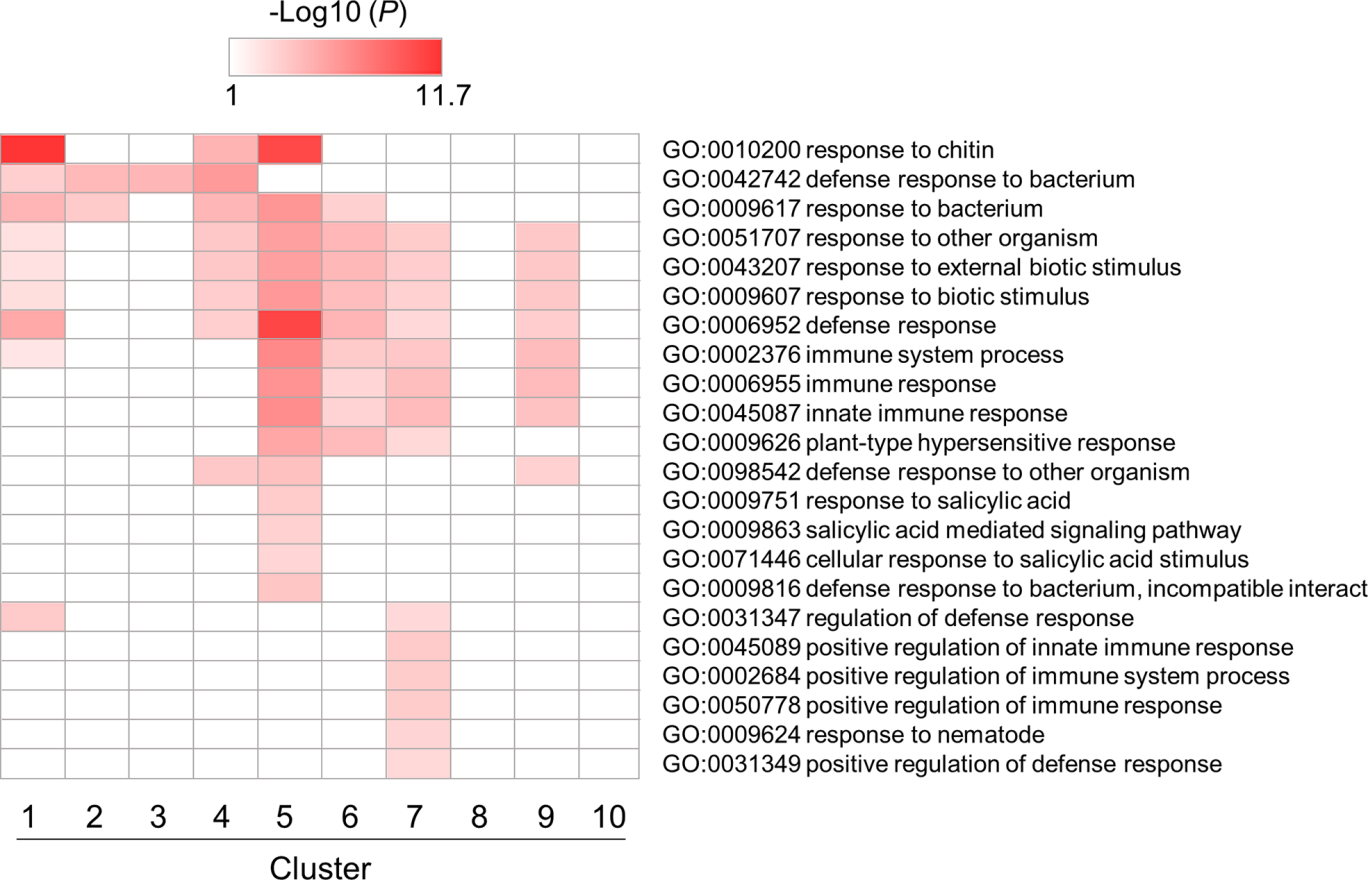
Gene ontology (GO) enrichment analysis of defense-related DEGs identified in flg22-treated *Brachypodium distachyon* leaf disks. The *p*-values of 22 GO terms in each cluster were analyzed, converted to logarithmic values (-Log10), and displayed as a heatmap, with darker colors indicating over-representation.

Next, we performed a GO enrichment analysis of a subset of DEGs involved in PTI in *B. distachyon* (**Supplementary Table S6-1–S6-10**). Among over-represented GO terms, enrichment patterns for GO terms related to defense responses were depicted in **Figure 4**. Most GO terms were enriched in clusters 5, 6, 7, and 9, which contained genes triggered by flg22. This result indicates that many genes categorized as defense-related genes were induced by flg22 within 3 h. On the other hand, some defense-related GO terms were enriched in clusters 1–4, which contained genes downregulated by flg22. This implies that these genes are involved in the negative regulation of defense responses. Alternatively but not mutually exclusively, the expression of these genes might be under feedback regulation to prevent the excessive response to MAMPs.

**Figure 5:**
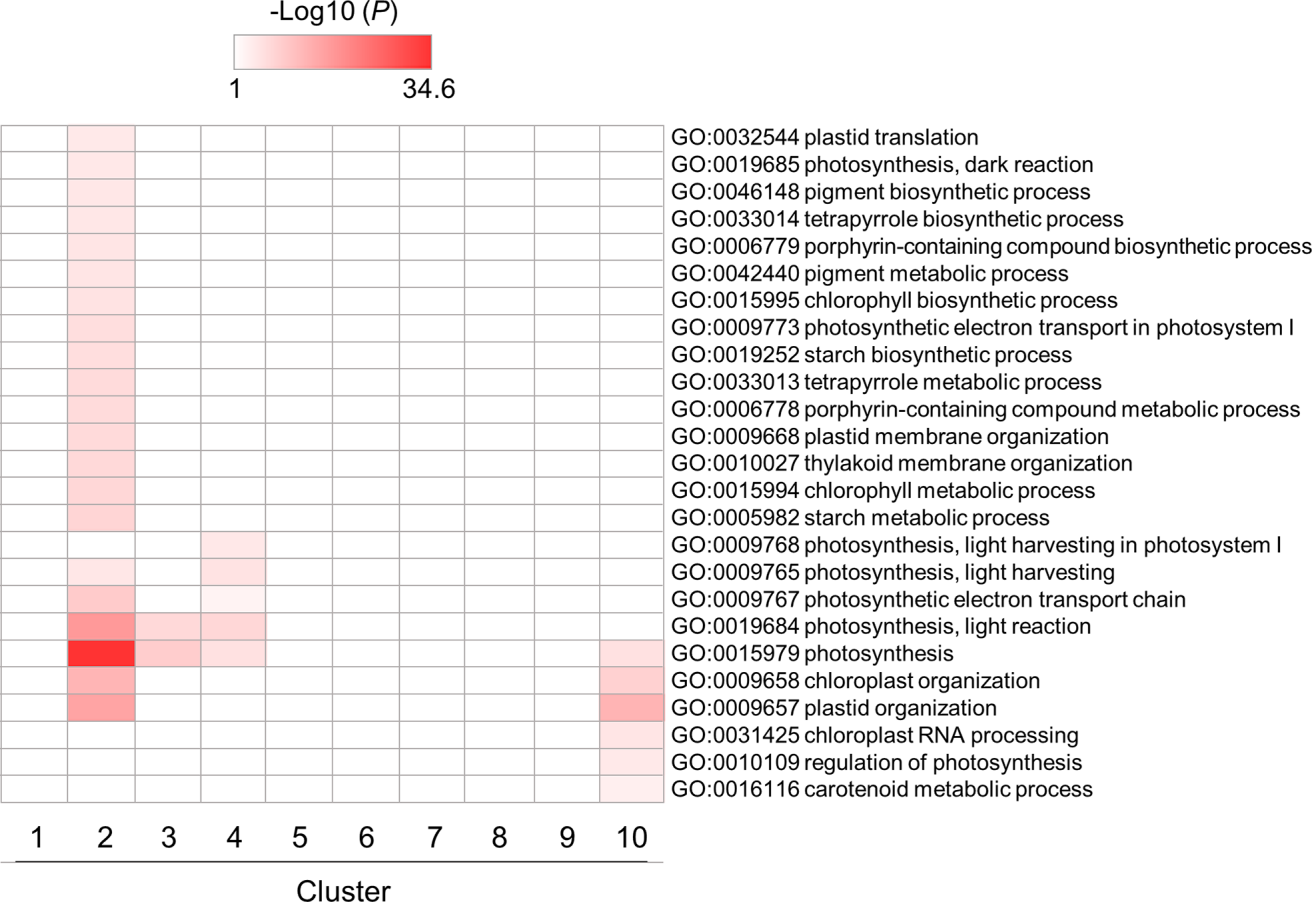
GO enrichment analysis of chloroplast- and photosynthesis-related DEGs identified in flg22-treated *Brachypodium distachyon* leaf disks. The *p*-values of 25 GO terms in each cluster were analyzed, converted to logarithmic values (-Log10), and represented as a heatmap, with darker colors indicating over-representation.

Enrichment patterns of GO terms related to photosynthesis and chloroplast are shown in **Figure 5**. Some GO terms were enriched in clusters 2, 3 and 4, which contained genes whose expression levels were downregulated by flg22, reaching almost minimum at 3 hat. This would be caused by a tradeoff between defense responses and photosynthesis in plants (Noutoshi et al., 2005; Bilgin et al., 2010; Göhre et al., 2012; Huot et al., 2014). In particular, the genes in cluster 2 showed temporal expression pattern (downregulated till 6 hat but back to the steady-state level at 12 hat). This result suggests that the chloroplast activities downregulated within 3 hat, because of the tradeoff, were reactivated from 12 hat onward. Other GO terms related to chloroplast activities, particularly RNA-related processes in chloroplast, were also enriched in cluster 10. This may reflect the recovery of chloroplast activities from defense. Otherwise, they may be simply as a result of the circadian rhythm.

### Enrichment analysis of TF genes and cis-elements in DEG promoters

**Figure 6:**
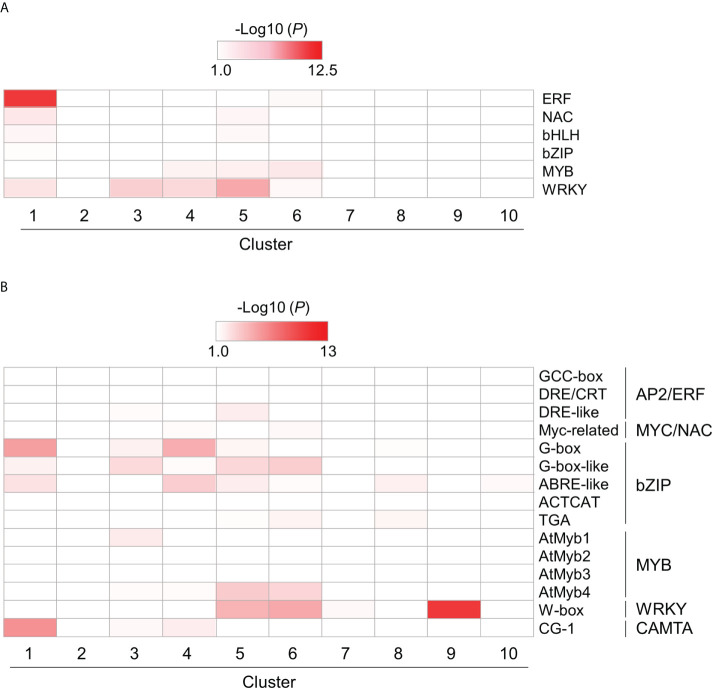
Over-representation analysis of selected DEGs encoding transcription factors (TFs) and harboring cis-regulatory elements in their promoter region. DEGs encoding TFs **(A)** and harboring cis-regulatory elements in their promoter region **(B)** were identified in flg22-treated *Brachypodium distachyon* leaf disks. The *p*-values of the listed TF-encoding and cis-regulatory motif-containing DEGs, which the selected TF could target, were analyzed in each cluster. The *p*-values were converted to logarithmic values (-Log10) and displayed as a heatmap, with darker colors indicating over-representation.

To investigate the role of TF families in PTI, we also examined the enrichment of genes encoding plant immunity-related TFs, such as AP2/ERF, NAC, bHLH, bZIP, MYB, and WRKY, in each cluster using hypergeometric tests (**Supplementary Table S8**). All TF genes, except those encoding MYB proteins, were enriched in cluster 1 (**Figure 6A** and **Supplementary Table S8**). Except for those encoding bZIP proteins, these TF genes were also enriched in clusters 4 and 5 (**Figure 6A** and **Supplementary Table S8**). These results suggest that PTI initiation involves various TFs to quickly mount a defense response after the recognition of MAMPs. We next examined the enrichment of DEGs harboring cis-regulatory elements in promoter regions (1 kb upstream of the open reading frame [ORF]), such as CGG-box, DRE/CRT, and DRE-like for AP2/ERF; Myc-related for MYC/NAC; G-box, G-box-like, ABRE-like, ACTCAT, and TGA for bZIP; AtMyb1, AtMyb2, AtMyb3, and AtMyb4 for MYB; W-box for WRKY; and CG-1 element for CALMODULIN-BINDING TRANSCRIPTIONAL ACTIVATOR (CAMTA) proteins (Phukan et al., 2016) (**Figure 6B** and **Supplementary Table S8**). Genes harboring bZIP-regulatory cis-elements were strongly enriched in clusters 1, 3, 4, 5, and 6. The results demonstrated in **Figure 4A** suggest that bZIP proteins could play positive and negative roles in gene transcription during PTI. The genes harboring cis-elements for MYC/NAC, MYB, and WRKY TFs in promoter regions were also enriched in clusters 5 and 6. The genes in clusters 5 and 6 may be regulated by NAC, MYB, and WRKY TFs.

Bjornson et al. (2021) demonstrated that *CAMTA1* is induced in Arabidopsis at 10 min after the MAMP treatment and is required for the induction of GSR to mount PTI. We identified seven *CAMTA*-like genes in *B. distachyon* through similarity searches (**Supplementary Table S7**; *Bradi1g21372*, *Bradi1g27170*, *Bradi1g60817*, *Bradi1g71810*, *Bradi2g59137*, *Bradi3g23800*, and *Bradi5g08167*), and one of these genes, *Bradi1g60817* (cluster 5), was potentially a functional homolog of Arabidopsis *CAMTA1*. Because the CG-1 element for CAMTAs was enriched in clusters 1, 3, and 4, which contained genes downregulated during PTI, CAMTAs may play a repressive role during PTI in *B. distachyon* at least 30 min after MAMP treatment.

### WRKY TFs potentially involved in PTI in *B. distachyon*

**Figure 7:**
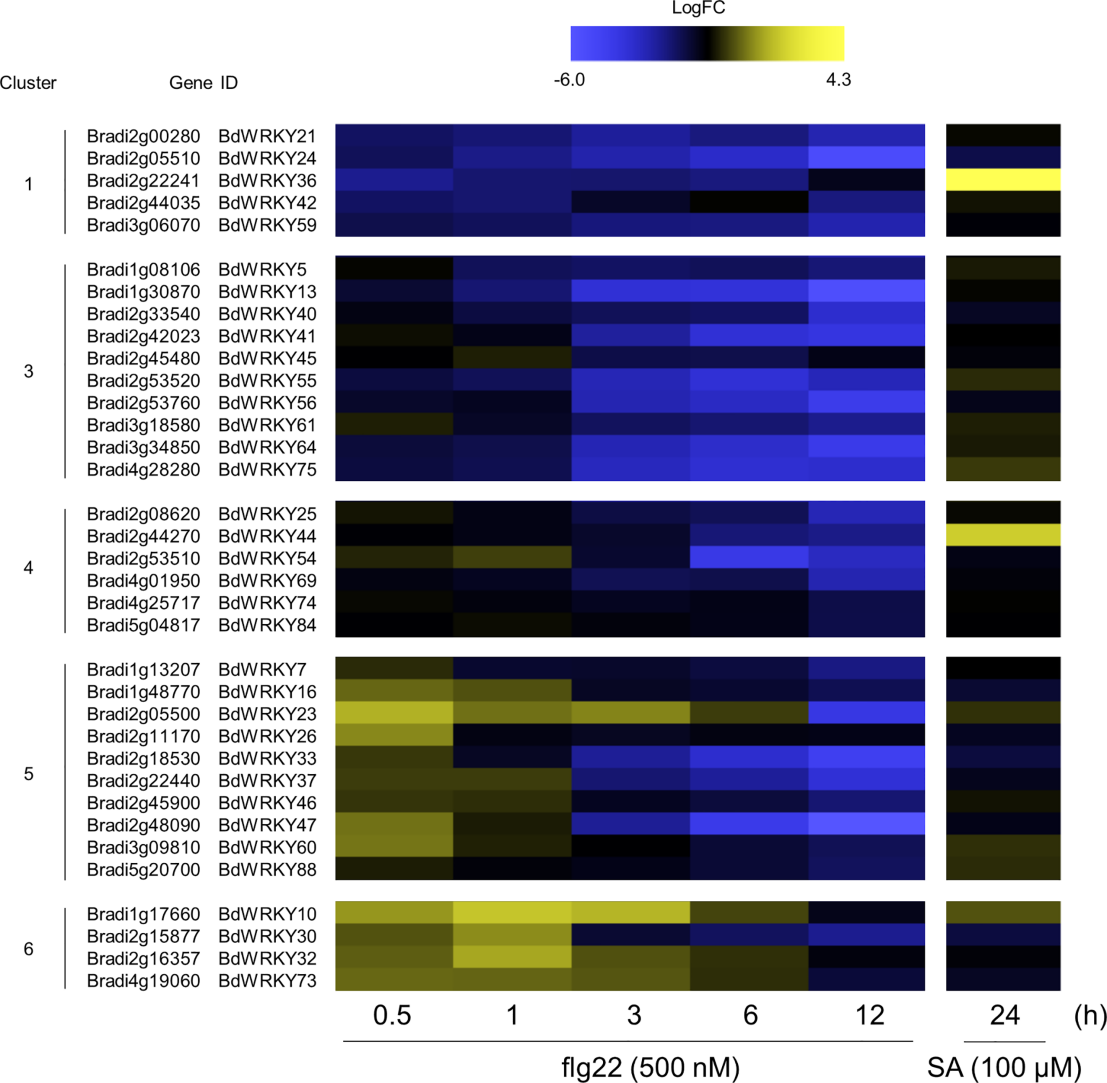
Expression patterns of WRKY TF-encoding DEGs identified in the flg22-treated *Brachypodium distachyon* leaf disks. Log2 (fold change) in the expression level of each *WRKY* gene at 0.5–12 h after flg22 treatment (left) and at 24 h after SA treatment (right; retrieved from Kouzai et al. (2020) compared with the non-treated control was calculated and represented as a heatmap. The higher expression values are shown in yellow color. Data are shown only for clusters 1, 3, 4, 5, and 6, in which significant over-representations of *WRKY* genes were detected.

WRKY TFs are known to perform positive and negative roles in defense responses, and the induction of many *WRKY* genes has been reported at 1 or 2 h after the flg22 treatment in *A. thaliana* (Birkenbihl et al., 2018). Our over-representation analysis demonstrated significant enrichment of *WRKY* genes in clusters 1, 3, 4, 5, and 6 (**Figure 6A**). Additionally, genes harboring the W-box cis-element in the promoter region were also enriched in clusters 5, 6, 7, and 9 (**Figure 6B**). These results strongly suggest the importance of *WRKY* genes in PTI in *B. distachyon*. The transcription profiles of *WRKY* genes in each cluster are displayed in **Figure 7**. Clusters 1, 3, 4, 5, and 6 contained 5, 10, 6, 10, and 4 *WRKY* genes, respectively. A total of 21 *WRKY* genes in clusters 1, 3, and 4 and 14 *WRKY* genes in clusters 5 and 6 were likely to be involved in PTI with positive and negative regulatory modes, respectively. This characteristic is consistent with previous reports showing that many Arabidopsis *WRKY* genes contribute to a regulatory signaling network at an early stage of PTI (Birkenbihl et al., 2018), and 34 *WRKY* genes were commonly induced at 10 and 30 min after treatments with various MAMPs in Arabidopsis (Bjornson et al., 2021).

**Figure 8:**
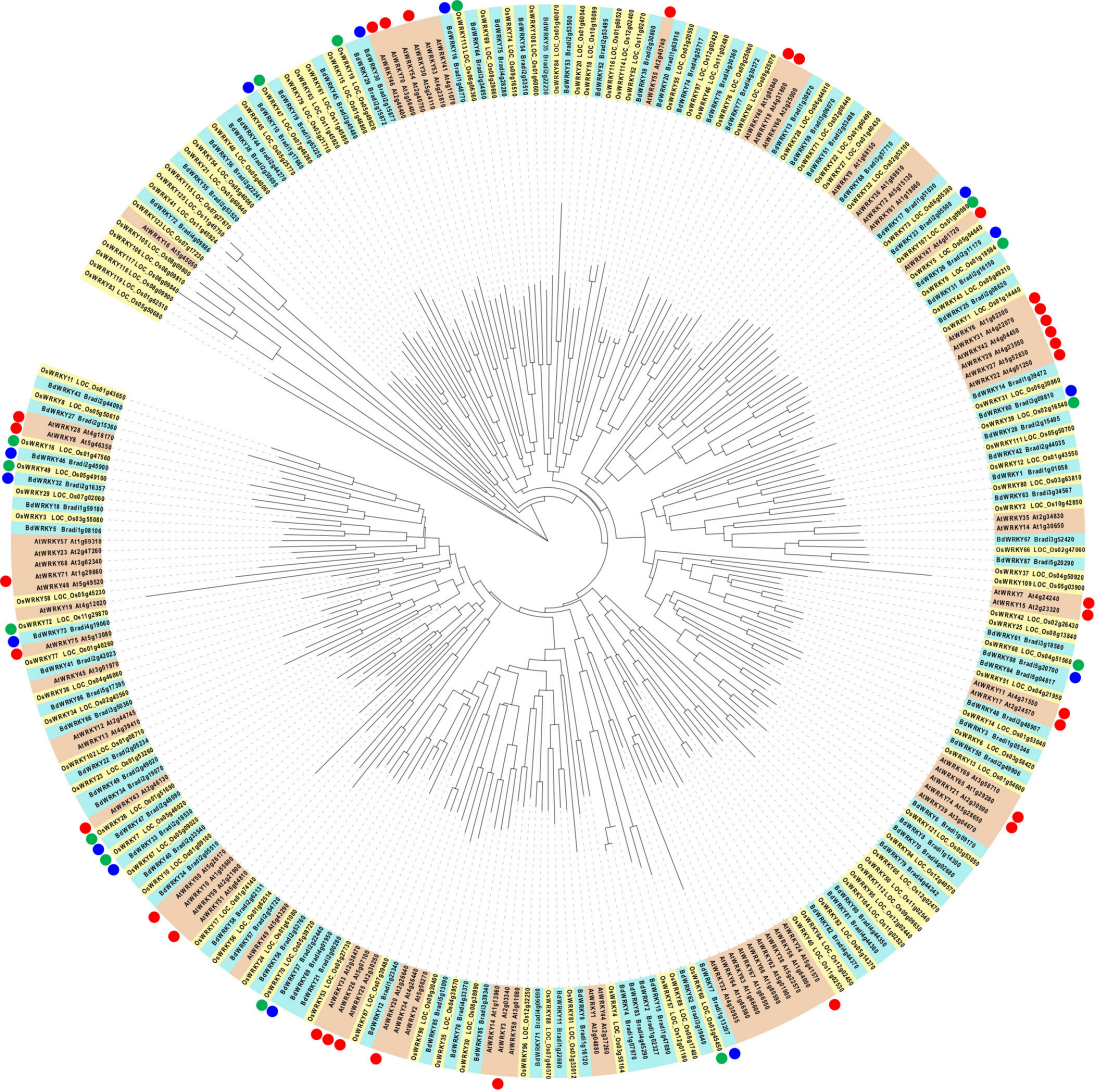
Phylogenetic analysis of WRKY proteins in Arabidopsis (red box), *Brachypodium distachyon* (blue box), and rice (yellow box), and their responsiveness to MAMPs. The nomenclatures of *WRKY* genes in Arabidopsis, rice, and (*B*) *distachyon* followed Eulgem et al. (2000); Rice WRKY Working Group (2012), and Kouzai et al. (2020), respectively. Arabidopsis *WRKY* genes, commonly induced at 30 min after the treatment of various MAMPs (Bjornson et al., 2021), are marked with red circles. Blue circles indicate (*B*) *distachyon WRKY* genes in clusters 5 and 6 (upregulated at 30 and 60 min after the flg22 treatment, respectively). Green circles indicate the rice homologs of (*B*) *distachyon WRKY* genes marked with blue circles.

We demonstrated that *BdWRKY38* and *BdWRKY44* act as master regulators of the salicylic acid (SA)-dependent defense response against *Rhizoctonia solani* (Kouzai et al., 2018; Kouzai et al., 2020). Phylogenetic analysis of the *WRKY* genes of Arabidopsis, rice, and *B. distachyon* (**Figure 8**) suggests that the encoded proteins of *BdWRKY38* and *BdWRKY44* are functional homologs of OsWRKY45 which plays a major role in the plant’s immune response. These two *WRKY* genes are strongly induced by SA (Kouzai et al., 2016). In this study, *BdWRKY38* was not included in the DEG list, whereas *BdWRKY44* was categorized in cluster 4. Further analyses are needed to confirm the functional contributions of the identified 35 *WRKY* genes in the DEGs and *BdWRKY38* in PTI in *B. distachyon*.

## Discussion

Cultured cells or calli have been frequently used to measure MAMP-triggered ROS production in rice because of their easy availability and maintenance. Few studies have described the assay using rice leaf disks; however, the signal intensity of cell suspension cultures appeared to be stronger than that of leaf disks (Kouzai et al., 2014a; Desaki et al., 2019). This study applied the ROS measurement method demonstrated in Arabidopsis to *B. distachyon* leaf disks (Albert et al., 2015). The ROS production pattern observed in response to MAMPs in *B. distachyon* was similar to that observed in Arabidopsis. In most of the previously published reports on ROS measurement in rice, luminol-based compounds were used to detect hydrogen peroxide (H_2_O_2_). We used a luminol derivative, L-012, to detect superoxide (O_2_^-^) in the current study. Additionally, using a luminescence microplate reader enabled the detection of ROS in real-time and with high sensitivity. Our results indicated that ROS measurement using leaf disks could work well in *B. distachyon*, as in Arabidopsis. We also demonstrated that *B. distachyon* senses chitin oligomer, flg22, and PGN as MAMPs, commonly used to monitor plant PTI. Overall, *B. distachyon* provides an alternative platform for studying immunity responses in monocotyledonous plants.

In rice, suspension cultures of cells and protoplasts derived from the cultivar Oc did not produce ROS in response to flg22 (Felix et al., 1999; Takai et al., 2007). In a subsequent study, cultured cells obtained from Oc and Kinmaze produced a low level of ROS at 0.5 h after the flg22 treatment (Takai et al., 2008). Rice protoplasts expressing *AtFLS2* produced ROS in response to flg22, and overexpression of *OsFLS2* in protoplasts prepared from the Arabidopsis *fls2* mutant complemented the flg22-induced ROS production (Takai et al., 2008). These results suggest that rice possesses a sensor molecule for flg22. In addition, the amount of ROS produced in response to flg22 was lower than that produced against flagellins purified from the *A. avenae* strain N1141 (Takai et al., 2008), indicating that rice exhibits low-level sensitivity to flg22, which varies with the expression level of *OsFLS2*. In cultured cells derived from the rice cultivar Nipponbare, the amount of H_2_O_2_ produced by flg22 was relatively lower than that produced by chitin or PGN (Kouzai et al., 2014a). By contrast, *B. distachyon* produced ROS after the recognition of flg22. Leaf disks of monocotyledonous plants, including wheat, barley, sorghum, and maize, were reported to produce ROS in response to flg22 (Proels et al., 2010; Zhang et al., 2017; Samira et al., 2020; Su et al., 2021), suggesting that these plant species recognize flg22 as a MAMP. The relative light unit (RLU) value in response to chitinheptaose was similar to that in response to flg22 (**Figure 1**), indicating that *B. distachyon* fully responds to flg22. Comparative analysis using full-length flagellin may clarify this point further and provide insight into another flagellin epitope.

In rice, OsFLS2 (LOC_Os04g52780; 1,174 amino acids [aa]) was identified as the closest homolog of AtFLS2 (At5g46330; 1,183 aa), with 45% sequence identity, based on homology search of the deduced amino acid sequences, and was shown to be functional (Takai et al., 2008). The protein annotated as BdFLS2 (Bradi5g21960; 1,223 aa) showed 46% and 67% sequence identity to AtFLS2 and OsFLS2, respectively, over an alignment length of 1,086 aa. The second closest *B. distachyon* homologs of OsFLS2 (35% sequence identity) were identified as Bradi1g26900 (1,151 aa alignment length), Bradi3g08070 (1,071 aa), Bradi1g37304 (997 aa), Bradi2g00540 (933 aa), and Bradi5g22620 (931 aa). BdFLS2 (Bradi5g21960) was categorized in cluster 5, and showed an increase in expression at 0.5 hat; however, the other second closest homologs were not identified in any of the clusters. Based on these observations, we speculate that BdFLS2 (Bradi5g21960) functions as an flg22 receptor.

Navarro et al. (2004) reported that *4CL*, *PAL2*, and *AtMYB2* were induced in cultured Arabidopsis cells at 30 and 60 min after flg22 treatment. Although no homologs of *PAL2* (*At3g53260*) and *AtMYB2* (*At2g47190*) have been identified in *B. distachyon*, *Bradi1g34300* was a counterpart of *4CL* (*At1g51680*). However, the *Bradi1g34300* gene was not induced in the current study and was not identified as a DEG. Among the 17 *Arabidopsis* orthologs of tobacco Avr9/Cf-9 rapidly elicited (*ACRE*) genes, 11 genes (*AtACRE1a*, *AtACRE1b*, *AtACRE31*, *AtACRE74a*, *AtACRE74b*, *AtACRE111*, *AtACRE126*, *AtACRE132*, *AtACRE264a*, *AtACRE276*, *AtACRE284a*) showed counterparts in *B. distachyon*. Among the 11 *B. distachyon* counterparts, *Bradi1g63480* (counterpart of *AtACRE31*; *At4g20780*, CML42) and *Bradi1g68530* (counterpart of *AtACRE74a*; *At5g37490*, ARM repeat superfamily protein) were found in cluster 5; *Bradi3g59830* (*AtACRE74b*; *At1g66160*, ATCMPG1) and *Bradi3g09000* (*AtACRE276*; *At1g29340*, ATPUB17) were found in cluster 6; and *Bradi1g51030* (*AtACRE264a*; *At2g05940*, protein kinase) was found in cluster 7, respectively. Notably, clusters 5–7 contained genes induced by flg22 within 3 h. The expression profiles of these *ACRE* genes in *A. thaliana* and *B. distachyon*, except *Bradi1g65520* (counterpart of *AtACRE284a*; *At2g30020*, protein phosphatase 2C [PP2C]), were similar.

In Arabidopsis, *FRK1* (*At2g19190*), *CYP81F2* (*At5g57220*), *NDR1/HIN1-LIKE 10* (*NHL10*; also known as *YLS9, At2g35980*), and *PHI-1* (*At1g35140*) were identified as marker genes for evaluating the activation of signaling pathways dependent on MAPK cascades and calcium-dependent protein kinases (Boudsocq et al., 2010). *Bradi2g55920*, the closest homolog of *NHL10* in *B. distachyon*, was found in cluster 1, which contained genes downregulated by flg22. *Bradi2g38960*, the closest homolog of Arabidopsis *FRK1*, was not identified as a DEG, and no counterparts of *CYP81F2* and *PHI-1* were identified in *B. distachyon*. Thus, the genes that serve as valuable markers in Arabidopsis are unavailable in *B. distachyon*. Because the expression profiles of genes do not necessarily overlap between *A. thaliana* and *B. distachyon*, the marker genes identified in this study can be used to study plant–microbe interactions in *B. distachyon*.

Our results suggested the involvement of NAC, MYB, WRKY, and CAMTA TFs in PTI. (Fujiwara et al., 2004) reported that *OsNAC3 (LOC_Os07g12340)*, *OsNAC4* (*LOC_Os01g60020*), *OsNAC6 (LOC_Os01g66120)*, and *WRKY (LOC_Os01g14440)* were induced in rice at 6 h after the flagellin treatment; however, the counterparts of these genes in *B. distachyon*, including *Bradi1g53770* (cluster 1), *Bradi2g53260* (cluster 4), *Bradi2g57100* (cluster 1), and *Bradi2g08620* (cluster 4), respectively, were not induced by flg22 in our study. This variation may be due to the difference in the experimental design of the two studies or materials between the two monocot species.

In Arabidopsis, 34 *WRKY* genes were commonly upregulated by various MAMPs within 30 min (Bjornson et al., 2021). In this study, 10 and 4 *WRKY* genes were identified in clusters 5 and 6, respectively, and were speculated to play a major role in inducing PTI in *B. distachyon*. In rice, *OsWRKY7*, *OsWRKY70*, *OsWRKY26*, *OsWRKY47*, and *OsWRKY19*, which are counterparts of *BdWRKY33*, *BdWRKY37*, *BdWRKY47*, *BdWRKY10*, and *BdWRKY30*, respectively, were reported to be induced by infection with rice blast fungus (Wei et al., 2013). This result suggests the conservation of the role of WRKYs in rice and *B. distachyon* during PTI.

Bjornson et al. (2021) identified 39 core immune response genes in Arabidopsis, including *GLUTAMATE RECEPTOR 2.9* (*GLR2.9*) and *GLR2.7*, and Arabidopsis *glr2.7 2.8 2.9* triple mutant plants showed reduced cytosolic calcium concentration in response to MAMPs and enhanced susceptibility to *Pseudomonas syringae* pv. *tomato*. In our transcriptome data, five *GLR* genes including four in cluster 5 (*Bradi4g30850*, *Bradi4g30860*, *Bradi4g30880*, and *Bradi3g53690*) and one gene in cluster 6 (*Bradi4g30840*), were found to be induced by flg22. Phylogenetic analysis suggested that Bradi4g30850 and Bradi4g30880 are the closest homologs of OsGLR1.2; Bradi4g30840 and Bradi4g30860 are the closest homologs of OsGLR1.1; and Bradi3g53690 is the closest homolog of OsGLR1.1 (**Supplementary Figure S2**). These genes belong to the GLR1 family, not the GLR2 family, unlike Arabidopsis; however, AtGLR2.7, 2.8, and 2.9 appeared adjacent to Bradi4g30850, Bradi4g30880, and OsGLR1.2, respectively, in the phylogenetic tree. Although we did not analyze the very early time points of PTI such as 5 or 10 min when *AtGLRs* are expressed, the GSR and its regulatory mechanism characterized in Arabidopsis are likely to be conserved in *B. distachyon*.

Genes identified in this study could serve as important candidates for the functional analysis of players involved in PTI not only in *B. distachyon* but also in other plant species.

## Data availability statement

The raw data of RNA-seq have been deposited in the DDBJ database [https://ddbj.nig.ac.jp/search] under BioSample accession numbers (Run accession numbers) SAMD00493633 (DRR378315), SAMD00493634 (DRR378316), SAMD00493635 (DRR378317) for control samples; SAMD00493636 (DRR378318), SAMD00493637 (DRR378319), and SAMD00493638 (DRR378320) for 0.5 h samples; SAMD00493639 (DRR378321), SAMD00493640 (DRR378322), and SAMD00493641 (DRR378323) for 1 h samples; SAMD00493642 (DRR378324), SAMD00493643 (DRR378325), and SAMD00493644 (DRR378326) for 3 h samples; SAMD00493645 (DRR378327), SAMD00493646 (DRR378328), and SAMD00493647 (DRR378329) for 6 h samples; and SAMD00493648 (DRR378330), SAMD00493649 (DRR378331), and SAMD00493650 (DRR378332) for 12 h samples. All data analyzed in this study are included in this article (Main figures and **Supplementary Files**).

## Author contributions

HM, MY, KTo, YI, and YN conceived of the study and designed the experiments. TO, YK, MW, and AT, carried out the ROS measurement and the qRT-PCR analyses. YK, KTa, J-SK, KM, and YN performed the RNA-seq analyses and data processing. YN drafted the manuscript and YK, HM, MY, KTo, YI, KM, and YN contributed to data interpretation and the critical revision of the manuscript. All authors contributed to the article and approved the submitted version.

## Funding

This research was supported by Grant-in-Aid for Scientific Research (KAKENHI) Grant Numbers 18H02206 and 21H02197 to YN from Japan Society for the Promotion of Science (JSPS).

## Acknowledgments

We would like to thank Dr. Shigeru Hanano (https://biopapyrus.jp/), Dr. Kazuma Uesaka (https://kazumaxneo.hatenablog.com/), and Dr. Ryuichiro Nakato (https://rnakato.hatenablog.jp/) for the kind introduction of basics and applications of a broad range of bioinformatics techniques on the web. For over-representation analysis, we referred to lecture slides provided by Dr. Koji Kadota https://www.iu.a.u-tokyo.ac.jp/~kadota/20180703_kadota_20180703.pdf). We also thank Dr. Atsushi Doi (http://array.cell-innovator.com/?p=431) for how to use MeV.

## Conflict of interest

The authors declare that the research was conducted in the absence of any commercial or financial relationships that could be construed as a potential conflict of interest.

## Publisher’s note

All claims expressed in this article are solely those of the authors and do not necessarily represent those of their affiliated organizations, or those of the publisher, the editors and the reviewers. Any product that may be evaluated in this article, or claim that may be made by its manufacturer, is not guaranteed or endorsed by the publisher.

## Supplementary material

Supplementary Table 6.2 Gene ontology (GO) enrichment analysis for cluster 2 of DEGs of *Brachypodium distachyon* during PTI (0, 0.5, 1, 3, 6, and 12 h) in response to flg22.

Supplementary Table 6.3 Gene ontology (GO) enrichment analysis for cluster 3 of DEGs of *Brachypodium distachyon* during PTI (0, 0.5, 1, 3, 6, and 12 h) in response to flg22.

Supplementary Table 6.4 Gene ontology (GO) enrichment analysis for cluster 4 of DEGs of *Brachypodium distachyon* during PTI (0, 0.5, 1, 3, 6, and 12 h) in response to flg22.

Supplementary Table 6.5 Gene ontology (GO) enrichment analysis for cluster 5 of DEGs of *Brachypodium distachyon* during PTI (0, 0.5, 1, 3, 6, and 12 h) in response to flg22.

Supplementary Table 6.6 Gene ontology (GO) enrichment analysis for cluster 6 of DEGs of *Brachypodium distachyon* during PTI (0, 0.5, 1, 3, 6, and 12 h) in response to flg22.

Supplementary Table 6.7 Gene ontology (GO) enrichment analysis for cluster 7 of DEGs of *Brachypodium distachyon* during PTI (0, 0.5, 1, 3, 6, and 12 h) in response to flg22.

Supplementary Table 6.8 Gene ontology (GO) enrichment analysis for cluster 8 of DEGs of *Brachypodium distachyon* during PTI (0, 0.5, 1, 3, 6, and 12 h) in response to flg22.

Supplementary Table 6.9 Gene ontology (GO) enrichment analysis for cluster 9 of DEGs of *Brachypodium distachyon* during PTI (0, 0.5, 1, 3, 6, and 12 h) in response to flg22.

Supplementary Table 6.10 Gene ontology (GO) enrichment analysis for cluster 10 of DEGs of *Brachypodium distachyon* during PTI (0, 0.5, 1, 3, 6, and 12 h) in response to flg22.

Supplementary Table 7
*Brachypodium distachyon* all genes with their closest homologs of *Arabidopsis thaliana* and *Oryza sativa*, annotations as transcription factors, and number of cis-elements in promoter region (1kbp).

Supplementary Table 8 Over-representation analysis of transcription factors (upper) and cis-elements (lower) in each DEG cluster of transcriptome analysis of *Brachypodium distachyon* in response to flg22 (used for ).

Supplementary Table 9 Primers used in this study.

## References

[B1] Albert M. Butenko M. A. Aalen R. B. Felix G. Wildhagen M. (2015). Chemiluminescence detection of the oxidative burst in plant leaf pieces. Bio-protocol 5, e1423. doi: 10.21769/BioProtoc.1423

[B2] Ao Y. Li Z. Feng D. Xiong F. Liu J. Li J. F.. (2014). OsCERK1 and OsRLCK176 play important roles in peptidoglycan and chitin signaling in rice innate immunity. Plant J. 80, 1072–1084. doi: 10.1111/tpj.12710 25335639

[B3] Bilgin D. D. Zavala J. A. Zhu J. Clough S. J. Ort D. R. DeLucia E. H. (2010). Biotic stress globally downregulates photosynthesis genes. Plant Cell Environ. 33, 1597–1613. doi: 10.1111/j.1365-3040.2010.02167.x 20444224

[B4] Birkenbihl R. P. Kracher B. Ross A. Kramer K. Finkemeier I. Somssich I. E. (2018). Principles and characteristics of the arabidopsis WRKY regulatory network during early MAMP-triggered immunity. Plant J. 96, 487–502. doi: 10.1111/tpj.14043 30044528

[B5] Bi G. Zhou Z. Wang W. Li L. Rao S. Wu Y.. (2018). Receptor-like cytoplasmic kinases directly link diverse pattern recognition receptors to the activation of mitogen-activated protein kinase cascades in arabidopsis. Plant Cell 30, 1543–1561. doi: 10.1105/tpc.17.00981 29871986 PMC6096590

[B6] Bjornson M. Pimprikar P. Nürnberger T. Zipfel C. (2021). The transcriptional landscape of *Arabidopsis thalia*na pattern-triggered immunity. Nat. Plants 7, 579–586. doi: 10.1038/s41477-021-00874-5 33723429 PMC7610817

[B7] Bolger A. M. Lohse M. Usadel B. (2014). Trimmomatic: A flexible trimmer for illumina sequence data. Bioinformatics 30, 2114–2120. doi: 10.1093/bioinformatics/btu170 24695404 PMC4103590

[B8] Boller T. Felix G. (2009). A renaissance of elicitors: perception of microbe-associated molecular patterns and danger signals by pattern-recognition receptors. Annu. Rev. Plant Biol. 60, 379–406. doi: 10.1146/annurev.arplant.57.032905.105346 19400727

[B9] Boudsocq M. Willmann M. R. McCormack M. Lee H. Shan L. He P.. (2010). Differential innate immune signalling *via* Ca^2+^ sensor protein kinases. Nature 464, 418–422. doi: 10.1038/nature08794 20164835 PMC2841715

[B10] Boutrot F. Zipfel C. (2017). Function, discovery, and exploitation of plant pattern recognition receptors for broad-spectrum disease resistance. Annu. Rev. Phytopathol. 55, 257–286. doi: 10.1146/annurev-phyto-080614-120106 28617654

[B11] Cai R. Lewis J. Yan S. Liu H. Clarke C. R. Campanile F.. (2011). The plant pathogen *Pseudomonas syringae* pv. *tomato* is genetically monomorphic and under strong selection to evade tomato immunity. PloS Pathog. 7, e1002130. doi: 10.1371/journal.ppat.1002130 21901088 PMC3161960

[B12] Cao Y. Liang Y. Tanaka K. Nguyen C. T. Jedrzejczak R. P. Joachimiak A.. (2014). The kinase LYK5 is a major chitin receptor in arabidopsis and forms a chitin-induced complex with related kinase CERK1. eLife 3, e03766. doi: 10.7554/eLife.03766 PMC4356144 25340959

[B13] Chambers J. P. Behpouri A. Bird A. Ng C. K. (2012). Evaluation of the use of the polyubiquitin genes, *Ubi4* and *Ubi10* as reference genes for expression studies in *Brachypodium distachyon* . PloS One 7, e49372. doi: 10.1371/journal.pone.0049372 23166649 PMC3498167

[B14] Chinchilla D. Zipfel C. Robatzek S. Kemmerling B. Nürnberger T. Jones J. D. G.. (2007). A flagellin-induced complex of the receptor FLS2 and BAK1 initiates plant defence. Nature 448, 497–500. doi: 10.1038/nature05999 17625569

[B15] Chisholm S. T. Coaker G. Day B. Staskawicz B. J. (2006). Host-microbe interactions: Shaping the evolution of the plant immune response. Cell 124, 803–814. doi: 10.1016/j.cell.2006.02.008 16497589

[B16] Clarke C. R. Chinchilla D. Hind S. R. Taguchi F. Miki R. Ichinose Y.. (2013). Allelic variation in two distinct *Pseudomonas syringae* flagellin epitopes modulates the strength of plant immune responses but not bacterial motility. New Phytol. 200, 847–860. doi: 10.1111/nph.12408 23865782 PMC3797164

[B17] Desaki Y. Shimada H. Takahashi S. Sakurayama C. Kawai M. Kaku H.. (2019). Handmade leaf cutter for efficient and reliable ROS assay. Plant Biotechnol. (Tokyo) 36, 275–278. doi: 10.5511/plantbiotechnology.19.0921a 31983882 PMC6978499

[B18] Dobin A. Davis C. A. Schlesinger F. Drenkow J. Zaleski C. Jha S.. (2013). STAR: Ultrafast universal RNA-seq aligner. Bioinformatics 29, 15–21. doi: 10.1093/bioinformatics/bts635 23104886 PMC3530905

[B19] Egusa M. Matsui H. Urakami T. Okuda S. Ifuku S. Nakagami H.. (2015). Chitin nanofiber elucidates the elicitor activity of polymeric chitin in plants. Front. Plant Sci. 6. doi: 10.3389/fpls.2015.01098 PMC4673310 26697049

[B20] Eulgem T. Rushton P. J. Robatzek S. Somssich I. E. (2000). The WRKY superfamily of plant transcription factors. Trends Plant Sci. 5, 199–206. doi: 10.1016/s1360-1385(00)01600-9 10785665

[B21] Felix G. Duran J. D. Volko S. Boller T. (1999). Plants have a sensitive perception system for the most conserved domain of bacterial flagellin. Plant J. 18, 265–276. doi: 10.1046/j.1365-313x.1999.00265.x 10377992

[B22] Fujiwara S. Tanaka N. Kaneda T. Takayama S. Isogai A. Che F. S. (2004). Rice cDNA microarray-based gene expression profiling of the response to flagellin perception in cultured rice cells. Mol. Plant-Microbe Interact. 17, 986–998. doi: 10.1094/MPMI.2004.17.9.986 15384489

[B23] Gentleman R. C. Carey V. J. Bates D. M. Bolstad B. Dettling M. Dudoit S.. (2004). Bioconductor: open software development for computational biology and bioinformatics. Genome Biol. 5, R80. doi: 10.1186/gb-2004-5-10-r80 15461798 PMC545600

[B24] Göhre V. Jones A. M. Sklenář J. Robatzek S. Weber A. P. (2012). Molecular crosstalk between PAMP-triggered immunity and photosynthesis. Mol. Plant-Microbe Interact. 25, 1083–1092. doi: 10.1094/MPMI-11-11-0301 22550958

[B25] Gust A. A. Biswas R. Lenz H. D. Rauhut T. Ranf S. Kemmerling B.. (2007). Bacteria-derived peptidoglycans constitute pathogen-associated molecular patterns triggering innate immunity in arabidopsis. J. Biol. Chem. 282, 32338–32348. doi: 10.1074/jbc.M704886200 17761682

[B26] Hayafune M. Berisio R. Marchetti R. Silipo A. Kayama M. Desaki Y.. (2014). Chitin-induced activation of immune signaling by the rice receptor CEBiP relies on a unique sandwich-type dimerization. Proc. Natl. Acad. Sci. U. S. A. 111, E404–E413. doi: 10.1073/pnas.1312099111 24395781 PMC3903257

[B27] Hind S. R. Strickler S. R. Boyle P. C. Dunham D. M. Bao Z. O'Doherty I. M.. (2016). Tomato receptor FLAGELLIN-SENSING 3 binds flgII-28 and activates the plant immune system. Nat. Plants 2, 16128. doi: 10.1038/nplants.2016.128 27548463

[B28] Huot B. Yao J. Montgomery B. L. He S. Y. (2014). Growth-defense tradeoffs in plants: A balancing act to optimize fitness. Mol. Plant 7, 1267–1287. doi: 10.1093/mp/ssu049 24777989 PMC4168297

[B29] Jin J. P. Tian F. Yang D. C. Meng Y. Q. Kong L. Luo J. C.. (2017). PlantTFDB 4.0: Toward a central hub for transcription factors and regulatory interactions in plants. Nucleic Acids Res. 45, D1040–D1045. doi: 10.1093/nar/gkw982 27924042 PMC5210657

[B30] Jones J. D. G. Dangl J. L. (2006). The plant immune system. Nature 444, 323–329. doi: 10.1038/nature05286 17108957

[B31] Kadota Y. Sklenar J. Derbyshire P. Stransfeld L. Asai S. Ntoukakis V.. (2014). Direct regulation of the NADPH oxidase RBOHD by the PRR-associated kinase BIK1 during plant immunity. Mol. Cell 54, 43–55. doi: 10.1016/j.molcel.2014.02.021 24630626

[B32] Kaku H. Nishizawa Y. Ishii-Minami N. Akimoto-Tomiyama C. Dohmae N. Takio K.. (2006). Plant cells recognize chitin fragments for defense signaling through a plasma membrane receptor. Proc. Natl. Acad. Sci. U. S. A. 103, 11086–11091. doi: 10.1073/pnas.0508882103 16829581 PMC1636686

[B33] Kishimoto K. Kouzai Y. Kaku H. Shibuya N. Minami E. Nishizawa Y. (2010). Perception of the chitin oligosaccharides contributes to disease resistance to blast fungus *Magnaporthe oryzae* in rice. Plant J. 64, 343–354. doi: 10.1111/j.1365-313X.2010.04328.x 21070413

[B34] Kouzai Y. Kimura M. Watanabe M. Kusunoki K. Osaka D. Suzuki T.. (2018). Salicylic acid-dependent immunity contributes to resistance against *Rhizoctonia solani*, a necrotrophic fungal agent of sheath blight, in rice and *Brachypodium distachyon* . New Phytol. 217, 771–783. doi: 10.1111/nph.14849 29048113 PMC5765516

[B35] Kouzai Y. Kimura M. Yamanaka Y. Watanabe M. Matsui H. Yamamoto M.. (2016). Expression profiling of marker genes responsive to the defence-associated phytohormones salicylic acid, jasmonic acid and ethylene in *Brachypodium distachyon* . BMC Plant Biol. 16, 59. doi: 10.1186/s12870-016-0749-9 26935959 PMC4776424

[B36] Kouzai Y. Mochizuki S. Nakajima K. Desaki Y. Hayafune M. Miyazaki H.. (2014a). Targeted gene disruption of *OsCERK1* reveals its indispensable role in chitin perception and involvement in the peptidoglycan response and immunity in rice. Mol. Plant-Microbe Interact. 27, 975–982. doi: 10.1094/MPMI-03-14-0068-R 24964058

[B37] Kouzai Y. Nakajima K. Hayafune M. Ozawa K. Kaku H. Shibuya N.. (2014b). CEBiP is the major chitin oligomer-binding protein in rice and plays a main role in the perception of chitin oligomers. Plant Mol. Biol. 84, 519–528. doi: 10.1007/s11103-013-0149-6 24173912

[B38] Kouzai Y. Shimizu M. Inoue K. Uehara-Yamaguchi Y. Takahagi K. Nakayama R.. (2020). BdWRKY38 is required for the incompatible interaction of *Brachypodium distachyon* with the necrotrophic fungus *Rhizoctonia solani* . Plant J. 104, 995–1008. doi: 10.1111/tpj.14976 32891065 PMC7756360

[B39] Kunze G. Zipfel C. Robatzek S. Niehaus K. Boller T. Felix G. (2004). The n terminus of bacterial elongation factor tu elicits innate immunity in arabidopsis plants. Plant Cell 16, 3496–3507. doi: 10.1105/tpc.104.026765 15548740 PMC535888

[B40] Laluk K. Luo H. Chai M. Dhawan R. Lai Z. Mengiste T. (2011). Biochemical and genetic requirements for function of the immune response regulator BOTRYTIS-INDUCED KINASE1 in plant growth, ethylene signaling, and PAMP-triggered immunity in arabidopsis. Plant Cell 23, 2831–2849. doi: 10.1105/tpc.111.087122 21862710 PMC3180795

[B41] Li Z. Ao Y. Feng D. Liu J. Wang J. Wang H. B.. (2017). OsRLCK 57, OsRLCK107 and OsRLCK118 positively regulate chitin- and PGN-induced immunity in rice. Rice (NY) 10, 6. doi: 10.1186/s12284-017-0145-6 PMC5318303 28220451

[B42] Li B. Dewey C. N. (2011). RSEM:accurate transcript quantification from RNA-seq data with or without a reference genome. BMC Bioinf. 12, 323. doi: 10.1186/1471-2105-12-323 PMC3163565 21816040

[B43] Li L. Li M. Yu L. Zhou Z. Liang X. Liu Z.. (2014). The FLS2-associated kinase BIK1 directly phosphorylates the NADPH oxidase RbohD to control plant immunity. Cell Host Microbe 15, 329–338. doi: 10.1016/j.chom.2014.02.009 24629339

[B44] Liu B. Li J. F. Ao Y. Qu J. Li Z. Su J.. (2012). Lysin motif-containing proteins LYP4 and LYP6 play dual roles in peptidoglycan and chitin perception in rice innate immunity. Plant Cell 24, 3406–3419. doi: 10.1105/tpc.112.102475 22872757 PMC3462640

[B45] Liu T. Liu Z. Song C. Hu Y. Han Z. She J.. (2012). Chitin-induced dimerization activates a plant immune receptor. Science 336, 1160–1164. doi: 10.1126/science.1218867 22654057

[B46] Lu D. Wu S. Gao X. Zhang Y. Shan L. He P. (2010). A receptor-like cytoplasmic kinase, BIK1, associates with a flagellin receptor complex to initiate plant innate immunity. Proc. Natl. Acad. Sci. U. S. A. 107, 496–501. doi: 10.1073/pnas.090970510 20018686 PMC2806711

[B47] Miya A. Albert P. Shinya T. Desaki Y. Ichimura K. Shirasu K.. (2007). CERK1, a LysM receptor kinase, is essential for chitin elicitor signaling in arabidopsis. Proc. Natl. Acad. Sci. U. S. A. 104, 19613–19618. doi: 10.1073/pnas.0705147104 18042724 PMC2148337

[B48] Navarro L. Zipfel C. Rowland O. Keller I. Robatzek S. Boller T.. (2004). The transcriptional innate immune response to flg22. interplay and overlap with avr gene-dependent defense responses and bacterial pathogenesis. Plant Physiol. 135, 1113–1128. doi: 10.1104/pp.103.036749 15181213 PMC514144

[B49] Noutoshi Y. Ito T. Seki M. Nakashita H. Yoshida S. Marco Y.. (2005). A single amino acid insertion in the WRKY domain of the arabidopsis TIR-NBS-LRR-WRKY-type disease resistance protein SLH1 (sensitive to low humidity 1) causes activation of defense responses and hypersensitive cell death. Plant J. 43, 873–888. doi: 10.1111/j.1365-313X.2005.02500.x 16146526

[B50] Phukan U. J. Jeena G. S. Shukla R. K. (2016). WRKY transcription factors: molecular regulation and stress responses in plants. Front. Plant Sci. 7. doi: 10.3389/fpls.2016.00760 PMC4891567 27375634

[B51] Proels R. K. Oberhollenzer K. Pathuri I. P. Hensel G. Kumlehn J. Hückelhoven R. (2010). RBOHF2 of barley is required for normal development of penetration resistance to the parasitic fungus *Blumeria graminis* f. sp. *hordei* . Mol. Plant-Microbe Interact. 23, 1143–1150. doi: 10.1094/MPMI-23-9-1143 20687804

[B52] Quinlan A. R. Hall I. M. (2010). BEDTools: A flexible suite of utilities for comparing genomic features. Bioinformatics 26, 841–842. doi: 10.1093/bioinformatics/btq033 20110278 PMC2832824

[B53] Rao S. Zhou Z. Miao P. Bi G. Hu M. Wu Y.. (2018). Roles of receptor-like cytoplasmic kinase VII members in pattern-triggered immune signaling. Plant Physiol. 177, 1679–1690. doi: 10.1104/pp.18.00486 29907700 PMC6084675

[B54] Rice WRKY Working Group (2012). Nomenclature report on rice WRKY's - conflict regarding gene names and its solution. Rice (NY) 5, 3. doi: 10.1186/1939-8433-5-3 PMC3834489 24764503

[B55] Robinson M. D. McCarthy D. J. Smyth G. K. (2010). edgeR: A bioconductor package for differential expression analysis of digital gene expression data. Bioinformatics 26, 139–140. doi: 10.1093/bioinformatics/btp616 19910308 PMC2796818

[B56] Saeed A. I. Bhagabati N. K. Braisted J. C. Liang W. Sharov V. Howe E. A.. (2006). TM4 microarray software suite. Methods Enzymol. 411, 134–193. doi: 10.1016/S0076-6879(06)11009-5 16939790

[B57] Samira R. Kimball J. A. Samayoa L. F. Holland J. B. Jamann T. M. Brown P. J.. (2020). Genome-wide association analysis of the strength of the MAMP-elicited defense response and resistance to target leaf spot in sorghum. Sci. Rep. 10, 20817. doi: 10.1038/s41598-020-77684-w 33257818 PMC7704633

[B58] Segonzac C. Zipfel C. (2011). Activation of plant pattern-recognition receptors by bacteria. Curr. Opin. Microbiol. 14, 54–61. doi: 10.1016/j.mib.2010.12.005 21215683

[B59] Shimizu T. Nakano T. Takamizawa D. Desaki Y. Ishii-Minami N. Nishizawa Y.. (2010). Two LysM receptor molecules, CEBiP and OsCERK1, cooperatively regulate chitin elicitor signaling in rice. Plant J. 64, 204–214. doi: 10.1111/j.1365-313X.2010.04324.x 21070404 PMC2996852

[B60] Su Y. Chen Y. Chen J. Zhang Z. Guo J. Cai Y.. (2021). Effectors of *Puccinia striiformis* f. sp. *tritici* suppressing the pathogenic-associated molecular pattern-triggered immune response were screened by transient expression of wheat protoplasts. Int. J. Mol. Sci. 22, 4985. doi: 10.3390/ijms22094985 34067160 PMC8125866

[B61] Sun Y. Li L. Macho A. P. Han Z. Hu Z. Zipfel C.. (2013). Structural basis for flg22-induced activation of the arabidopsis FLS2-BAK1 immune complex. Science 342, 624–628. doi: 10.1126/science.1243825 24114786

[B62] Takai R. Isogai A. Takayama S. Che F. S. (2008). Analysis of flagellin perception mediated by flg22 receptor OsFLS2 in rice. Mol. Plant-Microbe Interact. 21, 1635–1642. doi: 10.1094/MPMI-21-12-1635 18986259

[B63] Takai R. Kaneda T. Isogai A. Takayama S. Che F. S. (2007). A new method of defense response analysis using a transient expression system in rice protoplasts. Biosci. Biotechnol. Biochem. 71, 590–593. doi: 10.1271/bbb.60526 17284833

[B64] Tavazoie S. Hughes J. D. Campbell M. J. Cho R. J. Church G. M. (1999). Systematic determination of genetic network architecture. Nat. Genet. 22, 281–285. doi: 10.1038/10343 10391217

[B65] Tibshirani R. Walther G. Hastie T. (2001). Estimating the number of clusters in a data set *via* the gap statistic. J. R. Statist. Soc B. 63, 411–423. doi: 10.1111/1467-9868.00293

[B66] Untergasser A. Cutcutache I. Koressaar T. Ye J. Faircloth B. C. Remm M.. (2012). Primer3–new capabilities and interfaces. Nucleic Acids Res. 40, e115. doi: 10.1093/nar/gks596 22730293 PMC3424584

[B67] Wan J. Zhang X. C. Neece D. Ramonell K. M. Clough S. Kim S. Y.. (2008). A LysM receptor-like kinase plays a critical role in chitin signaling and fungal resistance in arabidopsis. Plant Cell 20, 471–481. doi: 10.1105/tpc.107.056754 18263776 PMC2276435

[B68] Wei T. Ou B. Li J. Zhao Y. Guo D. Zhu Y.. (2013). Transcriptional profiling of rice early response to *Magnaporthe oryzae* identified OsWRKYs as important regulators in rice blast resistance. PloS One 8, e59720. doi: 10.1371/journal.pone.0059720 23544090 PMC3609760

[B69] Willmann R. Lajunen H. M. Erbs G. Newman M. A. Kolb D. Tsuda K.. (2011). Arabidopsis lysin-motif proteins LYM1 LYM3 CERK1 mediate bacterial peptidoglycan sensing and immunity to bacterial infection. Proc. Natl. Acad. Sci. U. S. A. 108, 19824–19829. doi: 10.1073/pnas.111286210 22106285 PMC3241766

[B70] Winkelmüller T. M. Entila F. Anver S. Piasecka A. Song B. Dahms E.. (2021). Gene expression evolution in pattern-triggered immunity within *Arabidopsis thaliana* and across brassicaceae species. Plant Cell 33, 1863–1887. doi: 10.1093/plcell/koab073 33751107 PMC8290292

[B71] Yamada K. Yamaguchi K. Shirakawa T. Nakagami H. Mine A. Ishikawa K.. (2016). The arabidopsis CERK1-associated kinase PBL27 connects chitin perception to MAPK activation. EMBO J. 35, 2468–2483. doi: 10.15252/embj.201694248 27679653 PMC5109243

[B72] Yamaguchi K. Yamada K. Ishikawa K. Yoshimura S. Hayashi N. Uchihashi K.. (2013). A receptor-like cytoplasmic kinase targeted by a plant pathogen effector is directly phosphorylated by the chitin receptor and mediates rice immunity. Cell Host Microbe 13, 347–357. doi: 10.1016/j.chom.2013.02.007 23498959

[B73] Zhang X. Valdés-López O. Arellano C. Stacey G. Balint-Kurti P. (2017). Genetic dissection of the maize (*Zea mays* l.) MAMP response. Theor. Appl. Genet. 130, 1155–1168. doi: 10.1007/s00122-017-2876-6 28289802

[B74] Zipfel C. Robatzek S. Navarro L. Oakeley E. J. Jones J. D. G. Felix G.. (2004). Bacterial disease resistance in arabidopsis through flagellin perception. Nature 428, 764–767. doi: 10.1038/nature02485 15085136

