# Supplementary material for: Time-series transcriptome of Brachypodium distachyon during bacterial flagellin-induced pattern-triggered immunity
Source: Front Plant Sci. 2022 Sep 15;13:1004184. doi: 10.3389/fpls.2022.1004184 (PMC9521188; doi:10.3389/fpls.2022.1004184)
Supplement: Supplementary file 1 [file DataSheet_1.pdf]

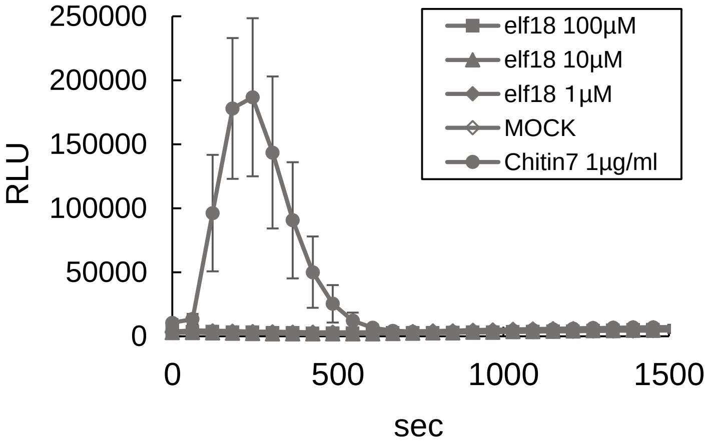

Supplementary Figure S1 | Analysis of reactive oxygen species (ROS) in the leaf disks of *Brachypodium distachyon* treated with elf18 peptide. Leaf disks of *B. distachyon* treated with elf18 peptide (1, 10 and 100 µM), chitinheptaose (1 µg/ml), and mock. Fluorescence was measured using a microplate reader and expressed as a relative light unit (RLU). Data represent means ± standard error (*n* = 4).

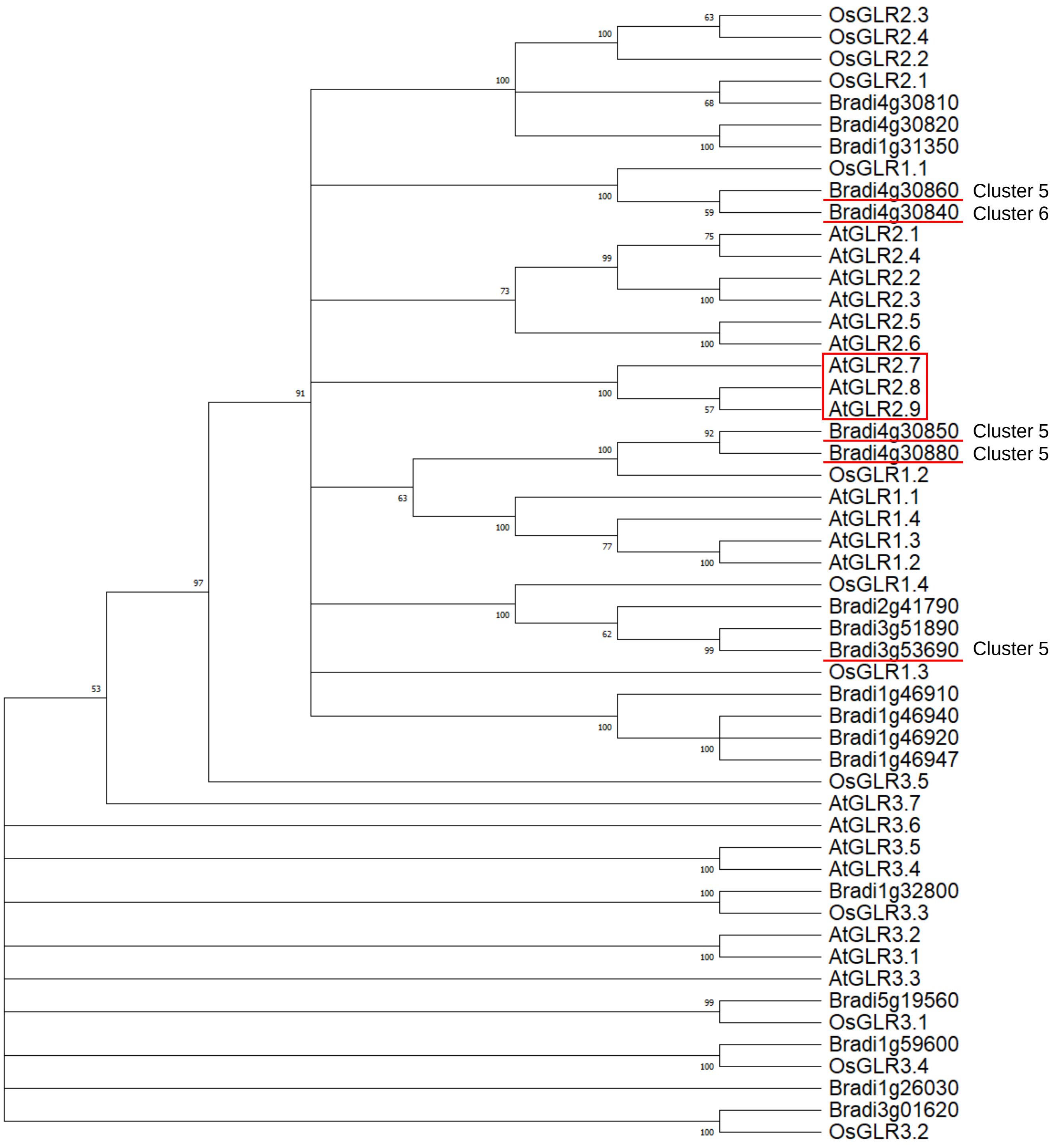

Supplementary Figure S2 | Evolutionary analysis of GLR proteins in *Arabidopsis thaliana*, *Oryza sativa* and *Brachypodium distachyon* by Maximum Likelihood method. The evolutionary history was inferred by using the Maximum Likelihood method and JTT matrix-based model. The bootstrap consensus tree inferred from 1000 replicates is taken to represent the evolutionary history of the taxa analyzed. Branches corresponding to partitions reproduced in less than 50% bootstrap replicates are collapsed. The percentage of replicate trees in which the associated taxa clustered together in the bootstrap test 1000 replicates)are shown next to the branches. Initial tree(s) for the heuristic search were obtained automatically by applying Neighbor-Join and BioNJ algorithms to a matrix of pairwise distances estimated using the JTT model, and then selecting the topology with superior log likelihood value. A discrete Gamma distribution was used to model evolutionary rate differences among sites (5 categories (+*G*, parameter = 2.0352)). The rate variation model allowed for some sites to be evolutionarily invariable ([+*I*], 2.50% sites). This analysis involved 52 amino acid sequences. All positions containing gaps and missing data were eliminated (complete deletion option). There were a total of 160 positions in the final dataset. Evolutionary analyses were conducted in MEGA11.

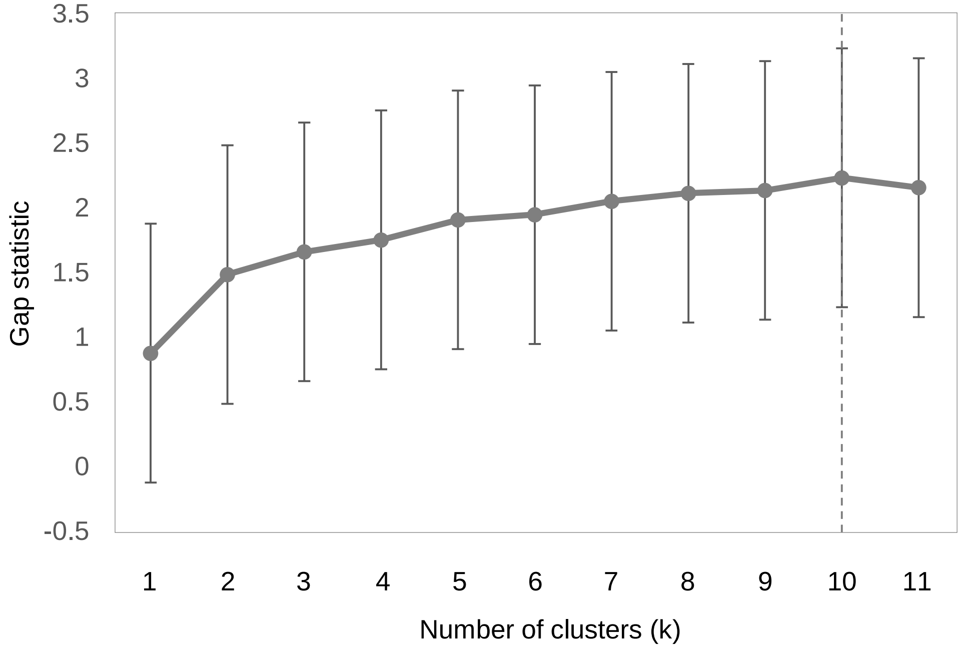

Supplementary Figure S3 | Result of gap statistic method. The optimal number of clusters in k-means clustering was determined for the classification of 6,059 differentially expressed genes (DEGs) identified by RNA-seq analysis of leaf disks with their expression patterns during 0–12 hours after treatment of flg22.
